# Agonistic antibacterial potential of *Loigolactobacillus coryniformis* BCH-4 metabolites against selected human pathogenic bacteria: An in vitro and in silico approach

**DOI:** 10.1371/journal.pone.0289723

**Published:** 2023-08-10

**Authors:** Anam Tariq, Mahwish Salman, Ghulam Mustafa, Abdul Tawab, Shazia Naheed, Hafsa Naz, Misbah Shahid, Hazrat Ali

**Affiliations:** 1 Department of Biochemistry, Government College University Faisalabad (GCUF), Faisalabad, Pakistan; 2 National Institute for Biotechnology and Genetic Engineering (NIBGE), Faisalabad, Pakistan; 3 Department of Applied Chemistry, Government College University Faisalabad (GCUF), Faisalabad, Pakistan; 4 Industrial Biotechnology Division, National Institute for Biotechnology and Genetic Engineering College, Pakistan Institute of Engineering and Applied Sciences (NIBGE-C,PIEAS), Faisalabad, Pakistan; Universidad Autonoma de Chihuahua, MEXICO

## Abstract

Lactic acid bacteria are known to produce numerous antibacterial metabolites that are active against various pathogenic microbes. In this study, bioactive metabolites from the cell free supernatant of *Loigolactobacillus coryniformis* BCH-4 were obtained by liquid-liquid extraction, using ethyl acetate, followed by fractionation, using silica gel column chromatography. The collected F23 fraction effectively inhibited the growth of pathogenic bacteria (*Escherichia coli*, *Bacillus cereus*, and *Staphylococcus aureus*) by observing the minimum inhibitory concentration (MIC) and minimum bactericidal concentrations (MBC). The evaluated values of MIC were 15.6 ± 0.34, 3.9 ± 0.59, and 31.2 ± 0.67 μg/mL and MBC were 15.6 ± 0.98, 7.8 ± 0.45, and 62.5 ± 0.23 μg/mL respectively, against the above-mentioned pathogenic bacteria. The concentration of F23 fraction was varying from 1000 to 1.9 μg/mL. Furthermore, the fraction also exhibited sustainable biofilm inhibition. Using the Electrospray Ionization Mass Spectrometry (ESI-MS/MS), the metabolites present in the bioactive fraction (F23), were identified as phthalic acid, myristic acid, mangiferin, 16-hydroxylpalmatic acid, apigenin, and oleandomycin. By using in silico approach, docking analysis showed good interaction of identified metabolites and receptor proteins of pathogenic bacteria. The present study suggested *Loigolactobacillus coryniformis* BCH-4, as a promising source of natural bioactive metabolites which may receive great benefit as potential sources of drugs in the pharmacological sector.

## Introduction

*Escherichia coli*, *Staphylococcus aureus*, and *Bacillus cereus* are omnipresent pathogenic bacteria that survive under variety of environmental conditions [1, 2]. Being agents of numerous diseases in human being, their significance derives from their ability of posing various health problems in their host organisms [3]. Bacteria have varied nature of health threat potentials *e*.*g*. *E*. *coli* is a prominent cause of diarrhoea, enteritis, and urinary tract infections [4] while *S*. *aureus* is responsible for various types of infections like skin infection, joint and bone infection, pneumonia, infective endocarditis, and osteomyelitis [5]. Similarly, *B*. *cereus* is also a well-known foodborne pathogen and causes several types of gastrointestinal diseases like diarrheal and emetic (vomiting) syndrome [2].

The control of these pathogenic bacteria is usually attained by using antibiotic drugs such as gentamicin, ciprofloxacin, clindamycin, amikacin, erythromycin, and vancomycin [6]. However, bacterial resistance to antibiotics is one of the major problems for patients’ treatment and control of such infectious diseases. Because many pathogenic bacteria have the ability of developing resistance to antibiotics such as *E*. *coli* have shown resistance against ciprofloxacin [7] while *S*. *aureus* to methicillin [8].

Natural products specially derived from microbes have been reported as biologically active metabolites and important sources of natural antibacterial agents. A variety of such natural products have been exploited by pharmaceutical industry as potent antibiotics [9]. Hence there is great need to discover and recognize new and unique antibiotics, especially from natural sources [10].

Lactic acid bacteria (LAB) are facultative anaerobes that play significant roles in food, agriculture, pharmaceutical industries [11]. These bacteria have been predominantly used as probiotics because of their safe status [Generally Recognized As Safe (GRAS) or Qualified Presumption of Safety (QPS)] [12]. Moreover, biosynthetic abilities of LAB are not only limited to vitamins and amino acids synthesis [13]. They also produce various types of bioactive metabolites like organic acids, exopolysaccharides, bacteriocins, cyclic dipeptides, fatty acids, and phenolics [14–18]. These metabolites exhibit antibacterial potential against gram- positive and gram-negative pathogenic bacteria e.g., *S*. *aureus*, *Listeria innocua*, *E*. *coli*, and *Hafnia alvei* [19, 20]. Therefore, such metabolites are considered as an appropriate alternative for antibiotic treatment and a better pharmaceutical approach [21].

In the current study, we mainly focused on the extraction of natural antibacterial metabolites, produced in *Loigolactobacillus coryniformis* BCH-4. This bacterium is a facultative anaerobic species of lactic acid-producing class of bacteria and has previously been reported for the production of various broad spectrum bioactive metabolites like organic acids, cyclic dipeptides, bacteriocins, and reuterin (3-hydroxypropionaldehyde, 3-HPA) [17, 22]. Initially the antibacterial metabolites were extracted by solvent-based extraction using ethyl acetate. The extract was fractionated using column chromatography-based purification approach. The antibacterial determination of potential fractions was examined against *E*. *coli*, *S*. *aureus*, and *B*. *cereus*. The chemical profile of most active fraction was analysed and confirmed by ESI-MS/MS both in negative and positive ion mode. Besides, *in silico* molecular docking was also performed to evaluate the causative mechanism of resultant antibacterial metabolites (ligands) and respective proteins (receptors) of selected pathogenic bacterial species.

## Materials and methods

### Required chemicals

Chemicals, culture media and HPLC grade organic solvents used in this study [ethyl acetate, chloroform, methanol, dimethyl sulfoxide (DMSO), Ethanol], crystal violet, de Man, Rogosa & Sharpe (MRS) agar/broth and nutrient agar/broth were mainly purchased from Sigma-Aldrich, USA. Moreover, Silica gel (70–230 mesh size) and thin-layer chromatography (TLC) silica gel 60 F_254_, aluminium sheet (20 x 20 cm^2^) were purchased from Merck, Darmstadt, Germany.

### Selected microbes and growth conditions

Previously isolated *Loigolactobacillus coryniformis* BCH-4 strain (Accession No. KX388387) [23], preserved at -80 °C in 15% (v/v) glycerol, was re-cultured at 37 °C on De Man, Rogosa and Sharpe (MRS) agar medium under aerobic conditions. Indicator bacterial strains, *Escherichia coli* ATCC 25922, *Bacillus cereus* ATCC 7064, and *Staphylococcus aureus* ATCC 25923 were purchased from the American Type Culture Collection (ATCC, Rockville, MD, USA) and were grown on nutrient medium for 16 h at 37 °C.

### Production and extraction of secondary metabolites

Six litres of MRS broth medium (pH 6.4 ± 0.2) were seeded with fresh *Loig*. *coryniformis* BCH-4 culture (10% v/v) in the fermenter (BioFer-010, ICCC, Pakistan) at optimized conditions of 37 °C for 72 h under constant stirring of 120 rpm [18]. After incubation, the culture was centrifuged at 4,430 × g (Z326K, Hermle, Germany) for 10 min at 4 °C and subsequently filtered through 0.22 μm pore size filters (Advantec MFS, Inc., Japan). The prepared cell-free supernatant (CFS) was lyophilized by freeze-drying (Alpha 2–4 LSC basic, Christ, Germany). For the extraction of metabolites, the freeze dried CFS powder was mixed with 50 mL sterile distilled water, and then added ethyl acetate as extracting solvent, with the ratio of 3:1 (v/v); ethyl acetate: CFS). The extract was concentrated using a rota vapor (R-210, Buchi, Switzerland), under vacuum at 38 °C [24].

### Fractionation of secondary metabolites

For the fractionation of various metabolites, the concentrated dark brown ethyl acetate extract was packed on to silica gel column. Gradient solvent system of chloroform and methanol [75:25 to 25:75] was used, and concentration of methanol was gradually increased. Forty fractions were collected in 10 mL glass vials and their TLC was performed. The TLC plates were observed under a UV lamp (UVGL-58, Cambridge, UK) for visualization of metabolic compounds [25]. Furthermore, the solvents were evaporated, and the dried fractions were redissolved in DMSO for determination of antibacterial potential.

### Antibacterial activity of column fractions

Disk diffusion method was performed in triplicate for determination of antibacterial potential of collected fractions. For this purpose, 6mm diameter filter paper disks were formed by punch machine and sterilized by autoclave at 121 °C in a sealed bottle. Fractions were dissolved in DMSO (10 mg/100 μL) and 10 μL of each of these fractions was applied on sterilized filter paper disk. While DMSO was used as negative control. Pathogenic bacterial test strains (10^8^ CFU/mL) were spread over nutrient agar plates and fractions impregnated disks were placed on agar plates using forceps. The plates were incubated for 24 h at 37 °C. Antibacterial potential was determined as inhibition zones around disk by using a ruler in millimetres (mm) [26]. Inhibitory zones were calculated by using following formula: [26]

InhibitionZone(mm)=Diameterofgrowthinhibitionzonearoundthedisk(mm)-Diameterofthedisk(6mm)

### Minimum Inhibitory Concentration (MIC) and Minimum Bactericidal Concentration (MBC)

MIC value of F23 fraction was measured by 96- well micro-dilution method. Two-fold serial dilution of F23 fraction was prepared as 1000, 500, 250, 125, 62.5, 31.2, 15.6, 7.8, 3.9, and 1.9 μg/mL to evaluate minimum inhibitory concentration (MIC). The 140 μL from each dilution was pipetted to 96-well microtiter plate. Afterward, the plate was incubated at 37 °C for 24 h after adding 10 μL of bacterial culture in each well. The bacterial cultures of nutrient medium without addition of sample were used as negative control. Growth inhibition was determined by measuring absorbance at 600nm. Lower value of MIC indicates the minimum drug is required for inhibiting the pathogenic organisms. Additionally, minimum bactericidal concentration (MBC) was determined by sub-culturing the 10 μL of bacterial suspension from MIC results on nutrient agar plate and incubated at 37 °C for 24 h. MBC was considered as lowest concentration that did not display any bacterial growth. The experiment was performed in triplicate [27].

### Biofilm inhibition assay

The biofilm inhibitory effect of bioactive fraction (F23) was evaluated by following the method of Famuyide et al. (2019) [28] with slight modifications. Concisely, 10 μL inoculum of *E*. *coli*, *B*. *cereus*, and *S*. *aureus* (OD_600nm_ = 1.0) was pipetted to individual broth medium and incubated in static conditions at 37 °C for 6 h. After that, F23 fraction concentrations (0.5×, 2×, 4×, and 8× MIC) were added into the wells (96-well microtiter plate) and further incubated for 24 h at 37 °C, without shaking. The cultures were gently discarded, and wells were air dried in laminar flow. After drying, the wells were rinsed 3 times with phosphate-buffered saline (PBS) to remove free-floating cells and stained with 100 μL [0.1% (*w*/*v*)] crystal violet. After incubating for 15 min at room temperature, the dye was discarded, and wells were reputedly washed with distilled water and dried at 65 °C for 1h. Lastly, destaining of cells was done by using 95% (v/v) ethanol for 30 min and optical density (OD) was determined at 590 nm. The assay was performed in triplicate and biofilm inhibitory activity (%) was estimated using the following formula in accordance with OD of control (untreated wells).


$$Biofilm\;inhibition(\%)=\frac{\left(OD\;control-OD\;treatment\right)}{OD\;control}\times 100$$

### Time–kill assay

The inoculum of each bacterium (1 × 10^8^ CFU/mL) treated with different concentrations of F23 fraction (2X, 4X, and 8X MIC) was used to perform the time-kill assay. Moreover, the untreated bacteria used as negative control (without F23 fraction). The bacterial suspensions were incubated under shaking (150 rpm) at 37 °C. Aliquots of 100 μL of each bacterial culture from each treatment were pipetted out at time intervals of 0, 4, 8, 16, and 24 h, and spread on nutrient agar plates. The plates were incubated at 37 °C for 24 h prior to colony counting and assay was performed in triplicate [29].

### ESI-MS/MS of bioactive fraction

Among all collected fractions, F23 was analysed for identification of bioactive metabolites, using ESI-MS/MS (Thermo Scientific corporation, USA) [30]. The direct insertion method was used, at negative mode. The sample flow rate, temperature, and mass range were maintained at 7.8 μL/min, 283 °C, and *m/z* 50–2000, respectively. Other ionization parameters like sheath gas, auxiliary gas, capillary voltage, and capillary temperature were optimized for MS factors to achieve the best ionization and ensure the optimal signals of daughter and parent ion fragments of analytes. The analysis was performed using Xcalibur (Xcalibur 2.0.7) and structural interpretation was performed using Chem Draw (Chem Draw Pro 8.0) manually and the data was compared with previously published data.

### Comparative antibacterial activity of F23 and commercial oleandomycin

Based on ESI-MS/MS analysis it was observed that F23 fraction had a macrolide antibiotic, oleandomycin in addition with other metabolites. So, the antibacterial potential of F23 fraction was compared with commercial antibiotic oleandomycin (15 μg/disc) against pathogenic bacterial strains. The antibacterial potential of F23 fraction and commercial antibiotic oleandomycin were checked using the previously described method [26], antibacterial assay was performed in triplicate.

### Statistical analysis

All experiments (antibacterial activity, antibiofilm activity and time kill assay) were performed in triplicates (n = 3) mean ± standard deviation/ error. Statistical analysis was performed with Minitab 15 (Minitab Inc., State College, PA), using one-way analysis of variance (ANOVA) followed by Tukey’s HSD test.

### Molecular docking analysis

#### Retrieval of 3D structures of receptor proteins

The three-dimensional (3D) structures of dihydrofolate reductase (PDB ID: 3FYV) of *S*. *aureus*, while DNA polymerase III alpha subunit (PDB ID: 4JOM) of *E*. *coli* and putative deacetylase BC1534 (PDB ID: 2IXD) of *B*. *cereus* were retrieved from RCSB Protein Data Bank [31] and used for molecular docking studies.

#### Preparation of ligand library and receptor optimization

The chemical structures of the deduced compounds (*i*.*e*., phthalic acid, myristic acid, oleandomycin, 16-hydroxypalmitic acid, apigenin, and mangiferin were downloaded from PubChem database [32] and saved after energy minimization. For accurate docking analysis, the selected receptor proteins were optimized by removing water molecules, adding hydrogen atoms, 3D protonation, and energy minimization.

#### Molecular docking

PyRx software was used for exploring the interactions between selected ligand molecules and receptor proteins [33]. Most appropriate interactions and bindings between ligands and receptor proteins were selected based on best S-score, root mean squared deviation (RMSD) and energy validation rankings. Discovery studio was used to visualize interactions between best ligand and receptor protein [34].

## Results

### Fractionation and antibacterial resistance

**Fig 1:**
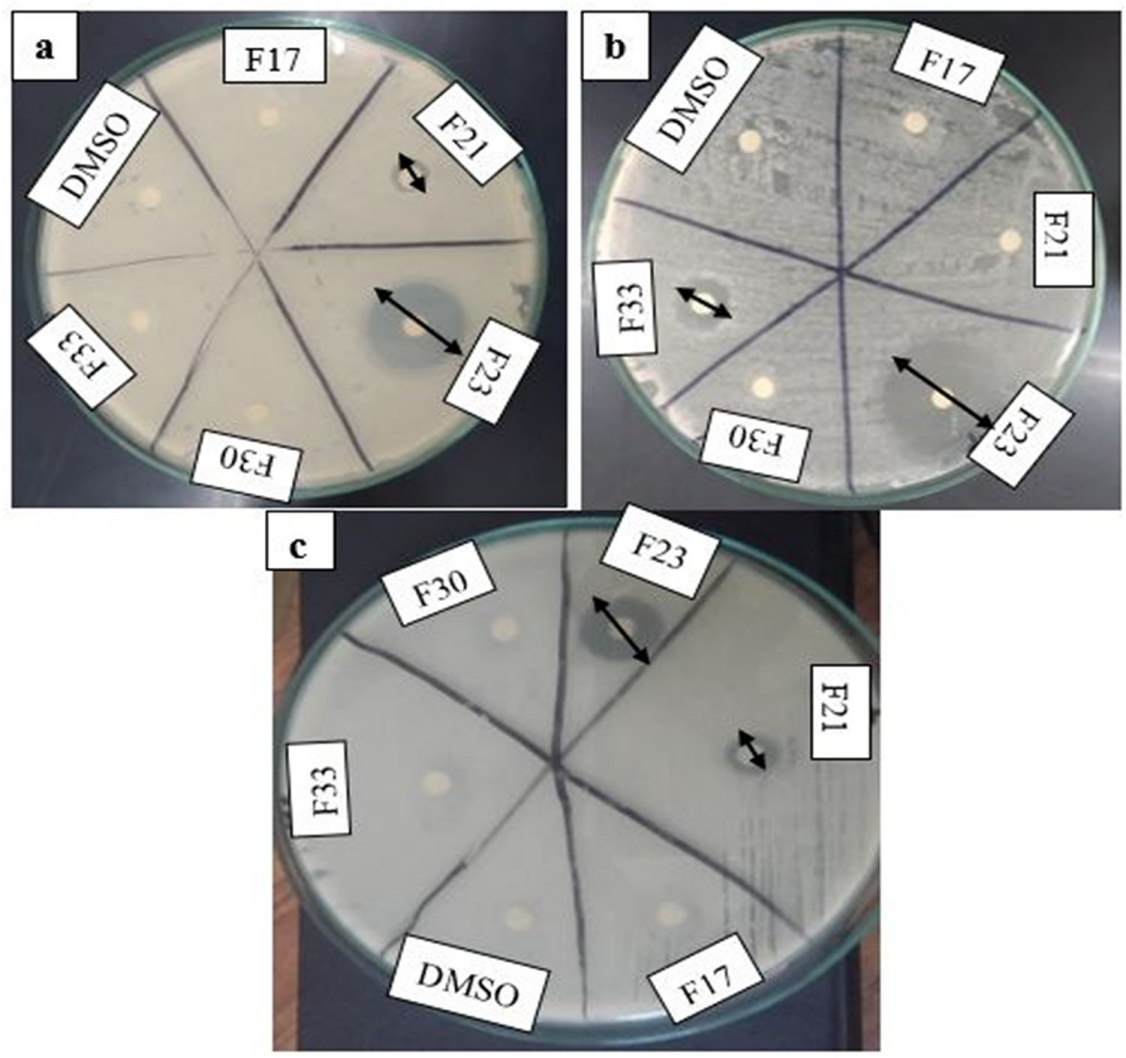
Antibacterial potential of column collected fractions (F17, F21, F23, F30, and F33) against a: *E*. *coli*,b: *B*. *cereus* and c: *S*. *aureus*, incubated at 37 °C for 24 h.

Five fractions (F17, F21, F23, F30, and F33) were selected after the visualization of clear component spots on the TLC plates, under ultraviolet radiations. The selected fractions were assayed for antibacterial activity. Fraction F23 eluted with CHCl_3_-MeOH (70: 30) gave the potent antibacterial activity as compared to other fractions (F17, F21, F30, and F33). The mean (n = 3) inhibition zones of bioactive fraction were 23.33 ± 0.57, 25.66 ± 0.57 and 19.33 ± 0.57 (mm) against *E*. *coli*, *B*. *cereus*, and *S*. *aureus* respectively (Fig 1). So, this (F23) fraction was selected for characterization of antibacterial metabolites. Moreover, the MIC values of this fraction were 15.6 ± 0.34, 3.9 ± 0.59, and 31.2 ± 0.67 (μg/mL) against *E*. *coli*, *B*. *cereus*, and *S*. *aureus* respectively. However, the MIC value against *B*. *cereus* (3.9 ± 0.59 μg/mL) was lower as compared to *E*. *coli* and *S*. *aureus* values.. Similarly, MBC values were 15.6 ± 0.98, 7.8 ± 0.45, and 62.5 ± 0.23 (μg/mL) against *E*. *coli*, *B*. *cereus*, and *S*. *aureus* respectively.

### Antibiofilm activity and time–kill assay

**Fig 2:**
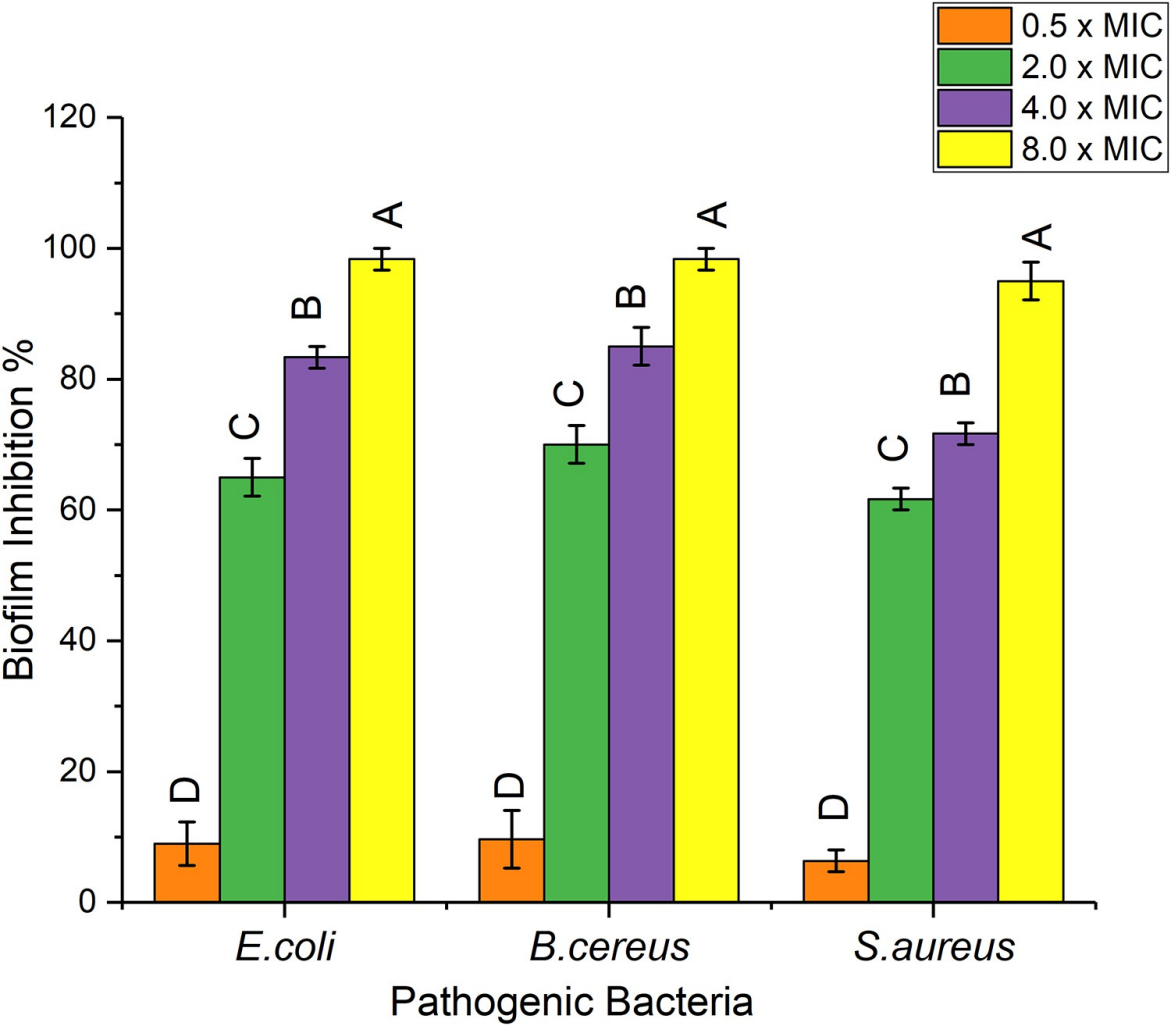
Anti biofilm potential of F23 fraction (0.5×, 2×, 4×, and 8× MIC) against pathogenic bacterial strains. Bars represent the standard error of mean (n = 3). Different letters indicate significant differences according to Tukey’s test with a *p*-value ≤0.05.

Biofilm formation was significantly (*p* < 0.001)inhibited by bioactive fraction (F23) against all selected pathogenic bacteria in dose dependent manner, as the formation of biofilm effectively decreased with increased concentration of F23 fraction (Fig 2). Moreover, lower concentration of F23 fraction exhibited lower biofilm inhibition percentage (below the MIC value for each pathogen).

**Fig 3:**
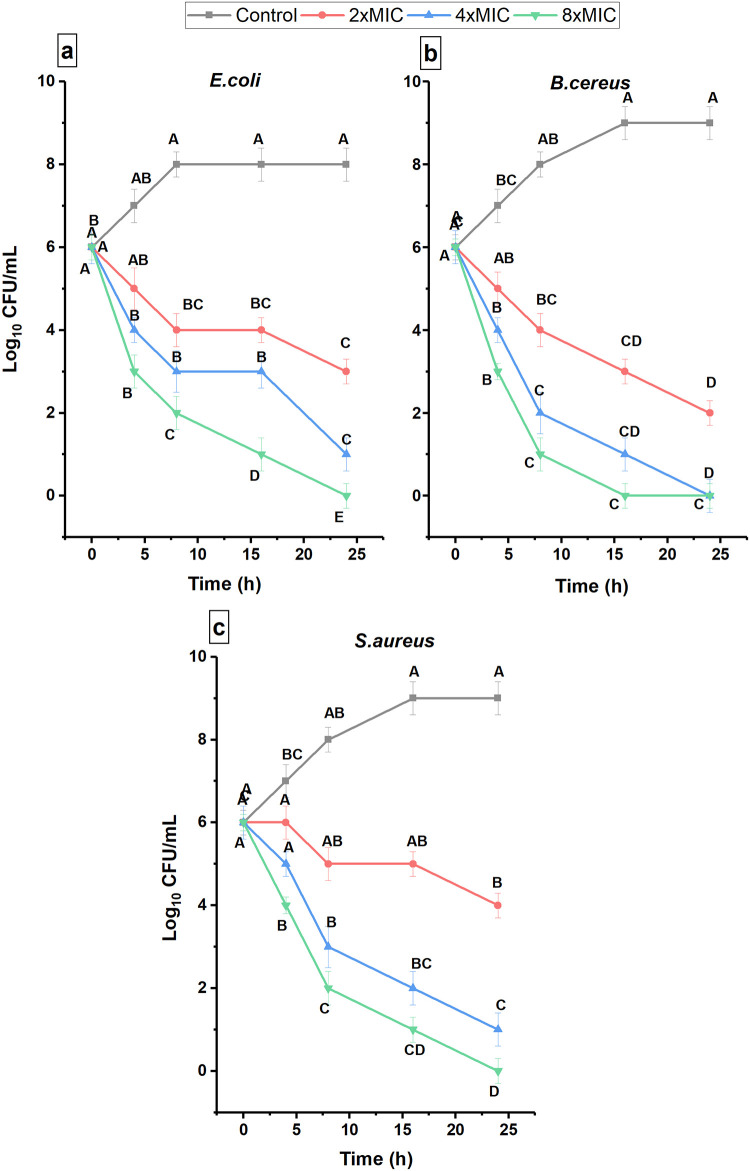
Time–kill assay of F23 fraction (0.5×, 2×, 4×, and 8× MIC) against (a) *E*. *coli*, (b) *B*. *cereus* and (c) *S*. *aureus* at different time periods (0, 4, 8, 16, and 24 h). Bars represent the standard error of the mean (n = 3). Results are according to ANOVA test mean values and intervals based on Tukey test. Different uppercase letters indicate significant differences according to the Tukey test with a *p*-value ≤0.05.

Time kill assay was performed over a period of 24 h with the pathogenic bacteria, being exposed to 2×, 4×, and 8× MIC of bioactive fraction. A graph was plotted between the logarithmic number of CFU/mL and time (Fig 3) which showed that bactericidal activity (bacteria completely killed) was observed at higher concentrations (4× and 8× MIC) after exposure of 24 h. Interestingly, *B*. *cereus* demonstrated a shorter time for the bactericidal effect at 8× MIC concentrations within 16 h.

### ESI-MS/MS analysis

To determine the probable bioactive compounds in fraction F23, it was subjected to tandem mass spectrometry. The ESI-MS/MS data of fraction F23 revealed the presence of various metabolites *i*.*e*., phthalic acid, myristic acid, mangiferin, 16-hydroxylpalmatic acid, apigenin and oleandomycin (Table 1). All these metabolites were identified based on their fragmentation patterns, during their electrospray ionization mass spectrometry.

**Table 1:** Metabolites of F23 fraction of *Loig*. *coryniformis* BCH-4, identified by ESI-MS/MS.

Sr. no.	Identified metabolites in fraction F23	Molecular mass	Ionization mode	Fragmentation	References
**1**	Phthalic acid	166.14	-ve	165, 147, 119, 73	[36]
**2**	Myristic acid	228.37	-ve	228, 184, 171, 156, 130, 114	[37]
**3**	16—hydroxypalmatic acid	272.42	-ve	271.1, 252.2, 239.0, 226.2, 213.1, 156.1	[38]
**4**	Apigenin	270.24	-ve	269.17, 251.17, 241.17, 225.1, 207.17, 197, 181, 155, 114	[39]
**5**	Mangiferin	422.33	-ve	421.25, 403.25, 389.33, 377.25, 349.17, 307.17, 291.17, 227.17	[40]
**6**	Oleandomycin	687.86	-ve	687.17, 669, 643.33, 597.33, 573.42, 521.25, 339.25, 325	[41]

**Fig 4:**
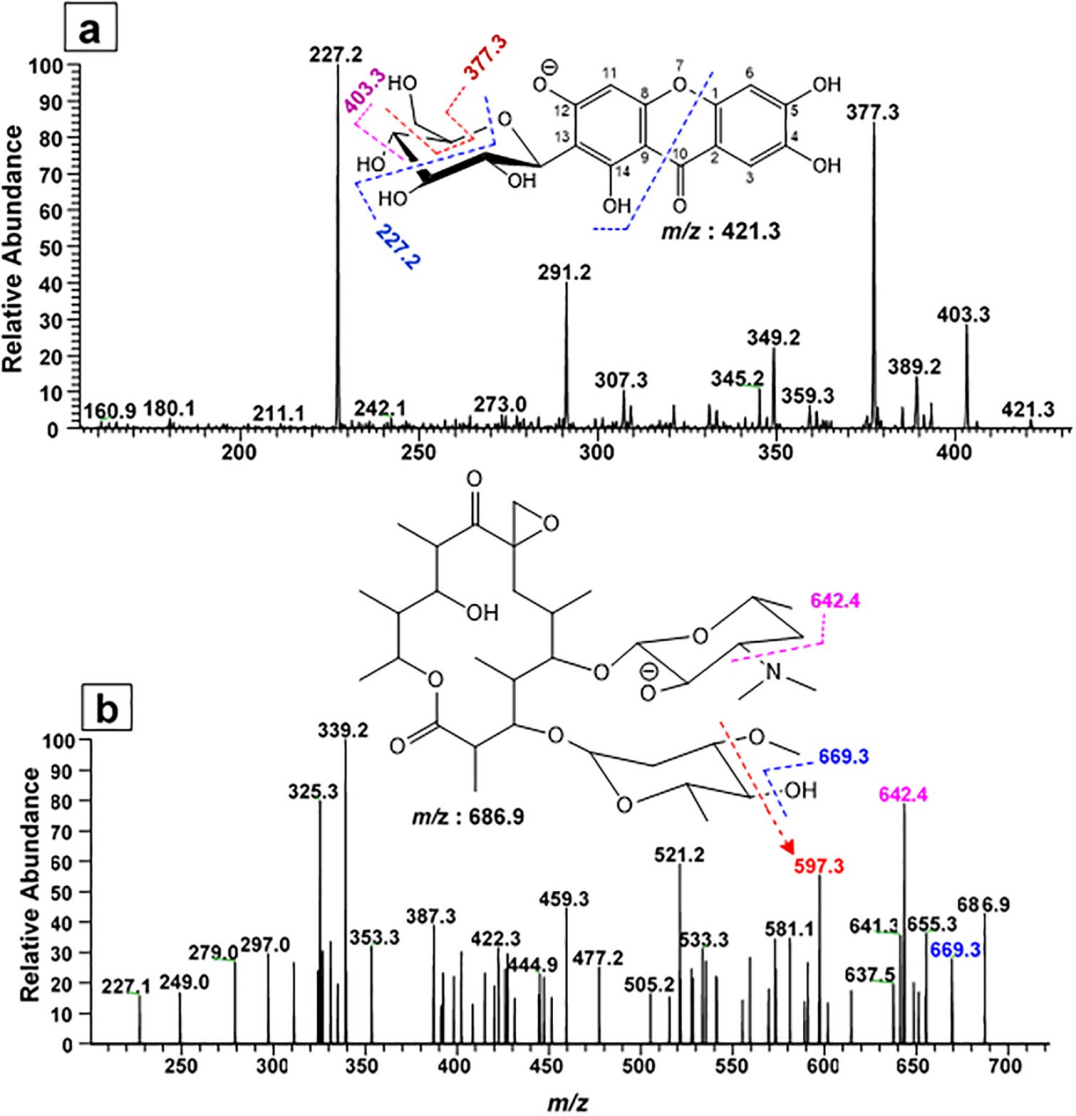
ESI-MS^2^ of metabolites (a) Mangiferin (*m/z* 421.3) @CID 5.30 and (b) Oleandomycin (*m/z* 686.9) @CID 10.0, in negative ion mode.

During the full ms, various ion peaks were obtained. These ion peaks were subjected to MS/MS. The full MS^2^ of ion peak at *m/z* 421.3 generated major daughter ion peaks at *m/z* 403.3, 389.2, 377.3, 349.2, 291.2 and 227.2. The ion peak at *m/z* 403.3 was due to the loss of water from the parent molecule. The loss of methoxy group from the C_5_ position of hexose resulted *m/z* 389.2. Similarly, the cross ring ^4,5^A_1_ fragmentation of hexose gave ion peak at *m/z* 377.3 while the ^3,5^A_1_ cross ring fragmentation [35] of hexose, along with C_2_-C_10_ and C_8_-O position of heterocyclic ring, generating the base ion peak at *m/z* 227.2, indicating the presence of mangiferin (Fig 4).

Oleandomycin is a microbial secondary metabolite and an antibiotic which was detected as ion peak of *m/z* 686.9. During its MS^2^, various fragment ions were detected. The ion peak at *m/z* 642.4 was obtained during the elimination of tertiary amine group from the hexose ring while the *m/z* 597.3 was due to the simultaneous loss of methoxy and hydroxyl groups (Fig 4).

During MS/MS, the ion peak at *m/z* 165.1 was also subjected to MS^2^, which generated the daughter ion peaks at *m/z* 147.1 and *m/z* 119.1 along with some other minor peaks. A base peak at *m/z* 147.1 was obtained due to the loss of water (165.1–147.1 = 18 mass units) from the molecular ion peak, converting it into phthalic anhydride, while the loss of carbonyl and rearrangement of phthalic anhydride generated propiolactone ring with ion peak at *m/z* 119.1 (S1 Fig in S1 File).

Similarly, the ion peak at *m/z* 227.4 generated daughter ion peaks of *m/z* 183.1, *m/z* 170.0, *m/z* 113.0 and a base peak of *m/z* 155.1. The *m/z* 113.0 was obtained due to the fragmentation of parent ion peak at C_6_ & C_7_, while the base peak was due to its cleavage at C_9_ and C_10_ position. Similarly, the cleavage at C_10_-C_11_ and C_11_-C_12_ generated daughter ion peaks at *m/z* 170.0 and *m/z* 183.1 respectively. This cleavage pattern reflected the parent ion peak at *m/z* 227.4 to be myristic acid (S2 Fig in S1 File).

During the MS^2^, another ion peak at *m/z* 271.2 fragmented into daughter ion peaks at *m/z* 254.2, 240.3, 227.2, 213.2 and a base ion peak at *m/z* 157.1. The base ion peak was obtained due to the fragmentation of the parent ion peak at C_9_-C_10_ position. The MS^2^ of 271.2 indicated hydroxyl (-OH) group best suiting at C_16_ position. In case of its docking at any other position, the *m/z* values of the daughter ion fragment below the *m/z* 254.2 should have one digit more than the existing pattern. This indicated the presence of 16-hydroxylpalmatic acid (S3 Fig in S1 File).

Another ion peak at *m/z* 269.1 was also detected. Its MS^2^ @CID 3.8, revealed its daughter ion peaks at *m/z* 253.2 due to water loss, *m/z* 237.0 due to loss of both hydroxyl groups and *m/z* 207.2 due to both hydroxyl loss along with carbonyl group of heterocyclic ring. The *m/z* 181.2, 155.1 were also produced during fragmentation. The base peak at *m/z* 225.2 was generated due to the loss of a carbonyl group from the C_7_ position of the heterocyclic ring along with one of the hydroxyl groups either from C_1_ or C_14_ position, indicating the presence of apigenin (S4 Fig in S1 File).

### Comparative antibacterial activity of F23 fraction and commercial oleandomycin

**Fig 5:**
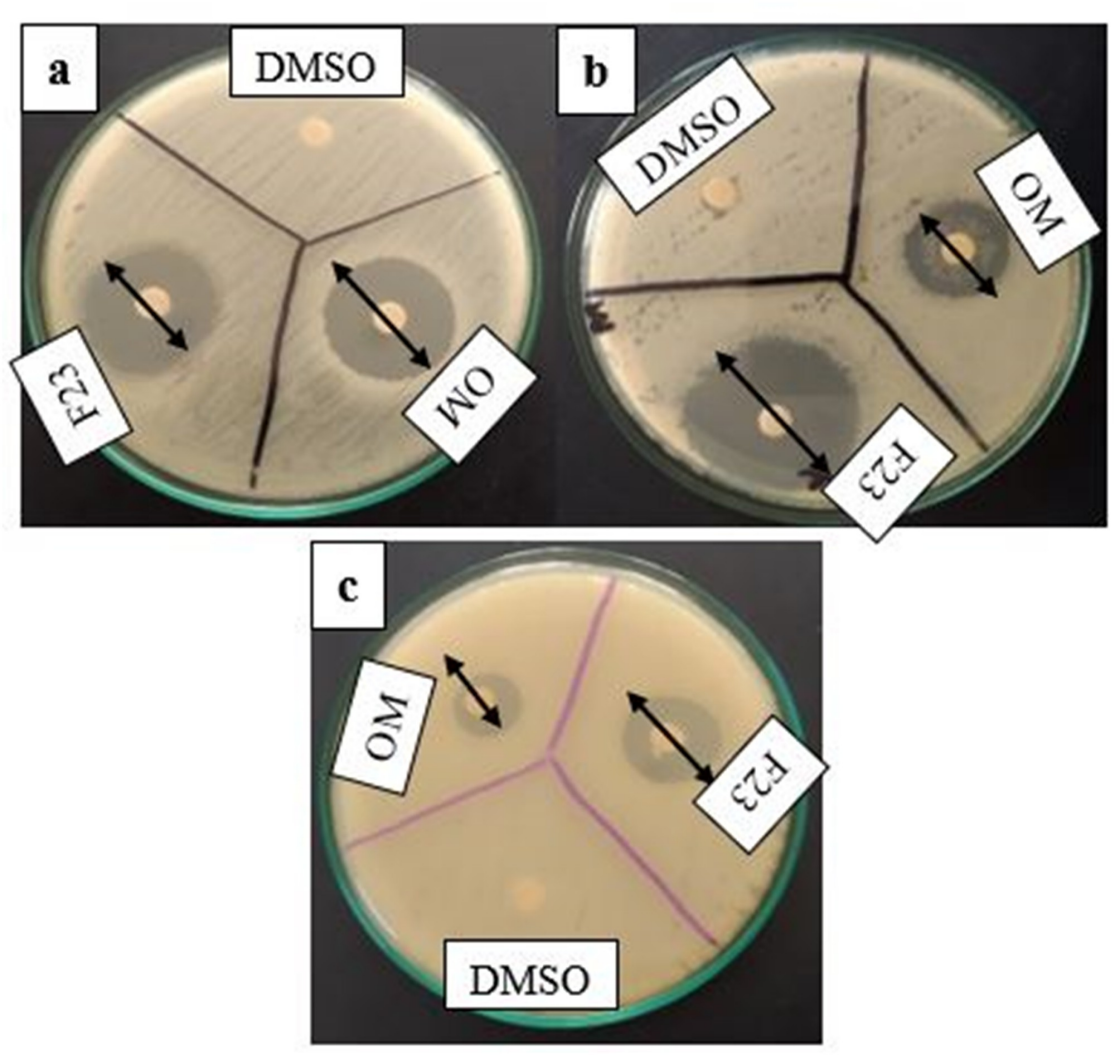
Antibacterial potential of F23 fraction and commercial oleandomycin (OM) antibiotic against. **a:** *E*. *coli*, **b:** *B*. *cereus* and **c:** *S*. *aureus* incubated at 37 °C for 24 h.

Antibacterial inhibitory zone of F23 fraction was 23.39 ± 0.57, 25.66 ± 0.57, and 19.30 ± 0.57 against *E*. *coli*, *B*. *cereus* and *S*. *aureus* respectively. But antibacterial activity of commercial oleandomycin was 21.45 ± 0.48, 19.45 ± 0.57, and 14.45 ± 0.24 against these strains (Fig 5). This fraction showed higher antibacterial activity against the tested pathogenic bacterial strains as compared to commercial oleandomycin antibiotic. It might be due to the agonistic effect of other metabolites *i*.*e*., phthalic acid, myristic acid, Mangiferin, 16-hydroxylpalmatic acid, apigenin, present in F23 fraction in addition with bioactive oleandomycin, and therefore contributed to enhance the antibacterial activity of F23 fraction.

### Molecular docking

Total six compounds were docked against receptor proteins of three selected bacteria, using PyRx software, to explore their antibacterial activities. The results showed the potency of selected compounds as good inhibitors of bacterial proteins. The best conformations were selected in each analysis, based on binding patterns and energy validations.

### Interaction analysis

**Fig 6:**
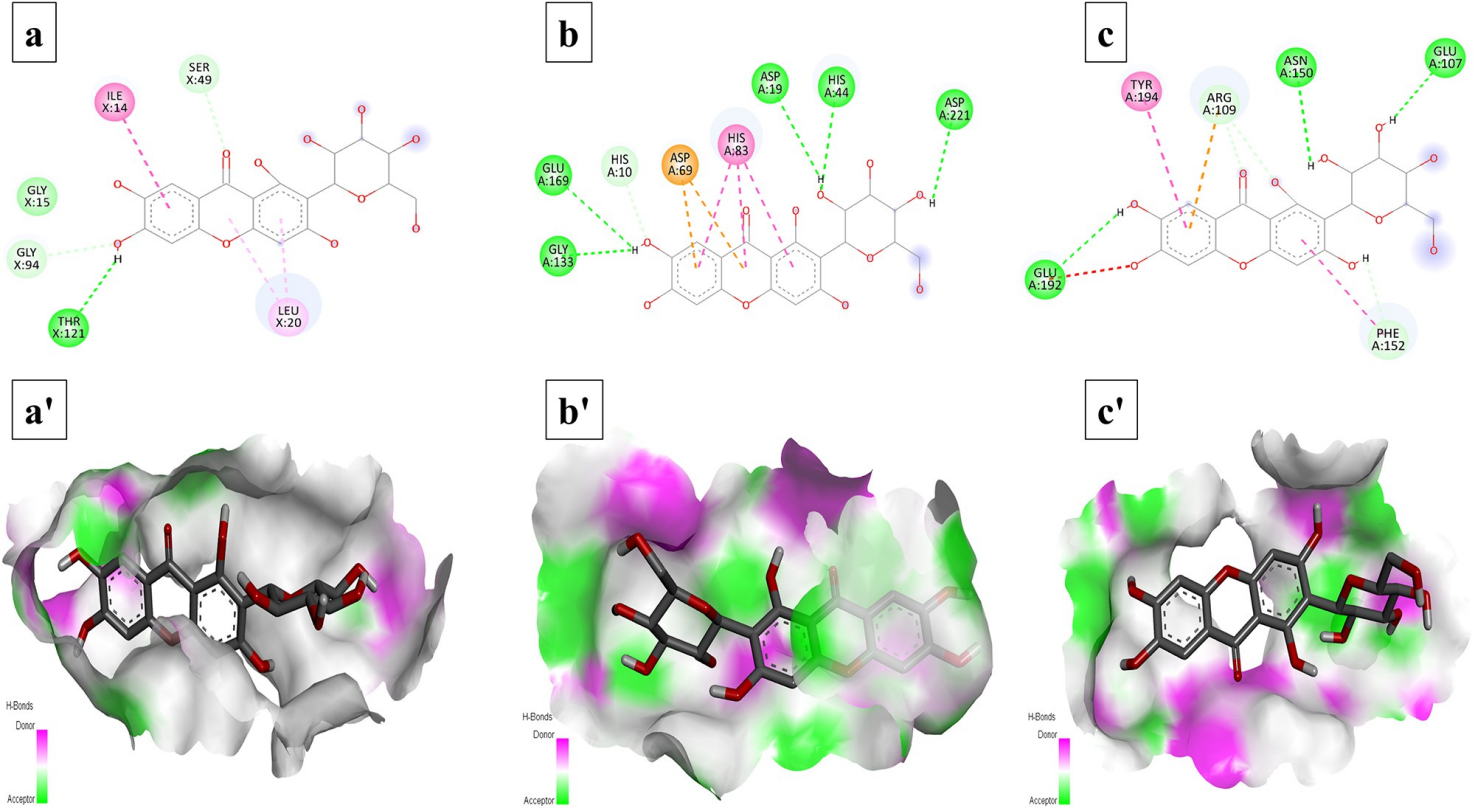
Interactions (a-c) and binding patterns (a’-c’) of Mangiferin with different receptor proteins. (a, a’) dihydrofolate reductase from *S*. *aureus* (b, b’) DNA polymerase III α-subunit from *E*. *coli* (c, c’) putative deacetylase BC1534 from *B*. *cereus*.

**Fig 7:**
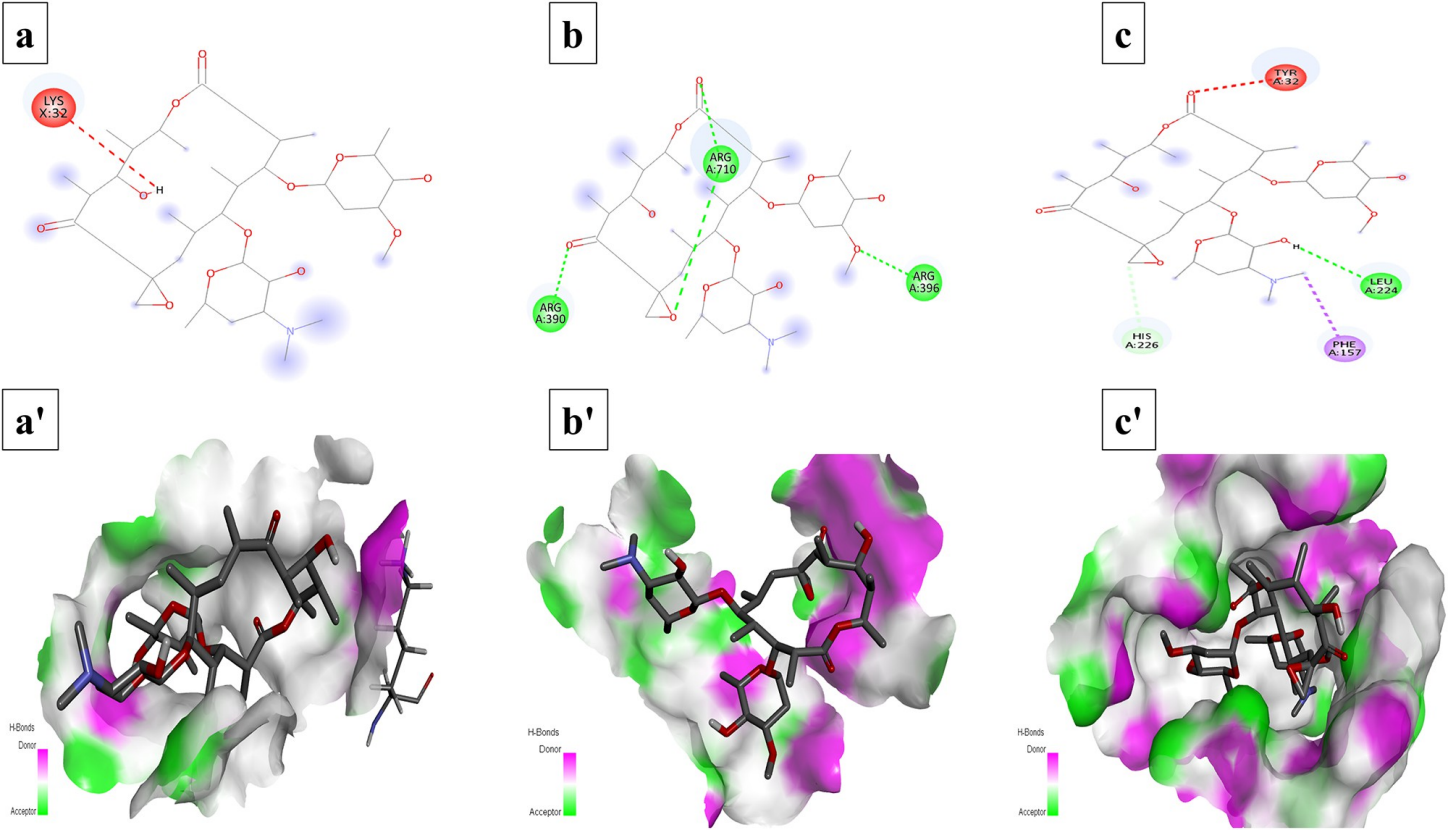
Interactions (a-c) and binding patterns (a’-c’) of oleandomycin with different receptor proteins. (a, a’) dihydrofolate reductase from *S*. *aureus* (b, b’) DNA polymerase III α-subunit from *E*. *coli* (c, c’) putative deacetylase BC1534 from *B*. *cereus*.

The conformations of ligands and bacterial proteins have been selected on the basis of their structural interactions and docking scores. The interactions of each bacterial proteins with these ligands have been given in Table 2, in descending order. Interestingly, mangiferin showed strong interactions with all three receptors with good S-score and RMSD values (Fig 6). Similarly, oleandomycin also exhibited strong interactions with all bacterial proteins (Fig 7). Both these compounds are showing their great potential to be used as antibacterial agents against selected bacteria. Phthalic acid (S5 Fig in S1 File), Myristic acid (S6 Fig in S1 File) and 16-hydroxypalmitic acid (S7 Fig in S1 File) revealed antibacterial activities against *S*. *aureus* and *E*. *coli* respectively. Moreover, Apigenin also showed good interaction with receptor proteins of selected bacteria (S8 Fig in S1 File).

**Table 2:** The interactions of metabolites (ligands) with receptor proteins of selected bacteria.

Sr. No.	Receptor protein	Source	Ligand	S-score	RMSD	Interacting residues
1	DNA polymerase III α-subunit	*E*. *coli*	Mangiferin	-9.1	0	His10, Asp19, His44, Asp69, His83, Gly133, Glu169, Asp221
			Oleandomycin	-7.4	0	Arg390, Arg396, Arg710
			Myristic acid	-4.7	0	Ser351, Lys352, Asn354, Gly355, Val358, Leu562, Ile565, Leu569, Ile586
			Apigenin	-9.7	0	His10, Asn71, Asp69, His83, Ala112, Gly133, Met136, Asp201
			Phthalic acid	-6.1	0	Asp69, Arg135, Met136, Arg175, Asp201
			16-hydroxypalmitic acid	-4.2	0	Lys352, Lys375, Asn566, Leu569, Ile586
2	Putative deacetylase BC1534	*B*. *cereus*	Oleandomycin	-7.4	0	Tyr32, Phe157, Leu224, His226
			Mangiferin	-7.0	0	Glu107, Arg109, Asn150, Phe152, Glu192, Tyr194
			Phthalic acid	-5.3	0	Ser46, Leu190
			Apigenin	-6.4	0	Ala42, Ser45, Ser46, Arg53, His110, Leu190
			16-hydroxypalmitic acid	-4.2	0	Phe157, Ile159, Val223
			Myristic acid	-4.4	0	Tyr27, Phe102, Tyr145, Phe157, Ile159, Phe217, Val223, Leu225, Asp228
3	Dihydrofolate reductase	*S*. *aureus*	Mangiferin	-7.3	0	Ile14, Gly15, Leu20, Ser49, Gly94, Thr121
			Oleandomycin	-7.3	0	Lys32
			Apigenin	-9.5	0	Gly43, Arg44, Lys45, Gly94, Thr96
			16-hydroxypalmitic acid	-6.1	0	Ala7, Asn18, Leu20, Lys45, Thr46
			Myristic acid	-5.8	0	Ala7, Leu20, Phe92
			Phthalic acid	-6.2	0	Ala7, Ile14, Asn18, Leu20, Thr46, Phe92

## Discussion

Due to the emergence of multidrug resistance (MDR) against various microbial pathogens, the search for new antimicrobial agents has been necessitated in recent years [42]. Antibiotic resistance is a problem that continues to challenge the healthcare sector worldwide, both in developing as well as developed countries [3, 43]. To combat the antibiotic resistant pathogens, beneficial microorganisms have been pursed due to their production of versatility of bioactive compounds which open new access to search compounds with therapeutic properties [9, 10]. Probiotic bacteria (lactic acid bacteria), which are also known as beneficial gut bacteria, have become a “popular therapy” in recent years [44].

The current work aimed to evaluate the bactericidal potential of *Loig*. *coryniformis* BCH-4 metabolites against human pathogenic bacterial strains. Antibacterial metabolites from *Loig*. *coryniformis* BCH-4 were extracted using ethyl acetate. The purpose for the use of this solvent was its effectiveness for extraction of bioactive metabolites as compared to other organic solvents (n- hexane and dichloromethane) [23]. This solvent has previously been used for extraction of various metabolites, produced by *B*. *subtilis* and *L*. *plantarum* [26, 45]. The crude extract of *Loig*. *coryniformis* BCH-4 was fractionated using silica gel chromatography. The eluted TLC visualized fractions were evaluated for their antibacterial potential against the three selected pathogenic bacterial strains *i*.*e*., *E*. *coli*, *B*. *cereus*, and *S*. *aureus*. The selection of these strains was based on their pathogenic nature and ubiquitous presence [46–48]. The F23 fraction of column chromatography showed maximum antibacterial potential as compared to all other fractions, against these three strains (Fig 1) with MIC: 15.6 ± 0.34, 3.9 ± 0.59 and 31.2 ± 0.67 (μg/mL) and MBC: 15.6 ± 0.98, 7.8 ± 0.45 and 62.5 ± 0.23 (μg/mL) against *E*. *coli*, *B*. *cereus*, and *S*. *aureus* respectively. The antibacterial potential of other *Lactobacillus* species *L*. *animalis*, *L*. *rhamnosus*, *L*. *fermentum*, *L*. *reuteri* have also been reported against *Yersinia enterocolitica* and *E*. *coli* [49]. Furthermore, bioactive compounds (organic acids, cyclic dipeptides and hydrogen peroxide) from *Lactobacillus* are widely used in food preservation and in biotechnology, and are being explored as therapeutics [50].

Biofilm formation is a clumping of bacterial groups together that firmly adhere to a solid surface surrounding by self- produced exopolysaccharides [27]. Its primary function is to protect the microorganisms from unfavourable conditions including resistance against antibacterial agents and host defence mechanisms [51]. Biofilm formation of pathogenic microorganisms helps them to attack host cells and continue the process of infection which is still a global threat for health due to its stubbornness of treatment and provoking ability to nosocomial infections [28].

In this work, the biofilm inhibition ability of F23 fraction was investigated, and it was observed that this fraction had excellent biofilm inhibitory potential against planktonic pathogenic bacteria (Fig 2). The formation of biofilm was decreased by increasing the concentration of F23. Furthermore, time kill assay of F23 fraction had bactericidal effect against selected bacterial pathogens by observing strong decrease in CFU/mL (Fig 3).

The ESI-MS/MS data revealed the presence of six bioactive compounds: phthalic acid, myristic acid, Mangiferin, 16-hydroxylpalmatic acid, apigenin and oleandomycin, in fraction 23 (Fig 4 & S1–S4 Figs in S1 File). This technique has previously been used for the identification of such compounds [52]. Phthalic acid identified from *Nonomuraea* species showed potent antibacterial potential against *Micrococcus luteus*, *B*. *substilis*, *S*. *epidermis*, *S*. *aureus*, *MRSA S*. *aureus*, *Klebsiella pneumonia*, *Enterobacter aerogens*, *Vibrio parahaemolyticus*, Yersinia *enterocolitica*, *Salmonella typhimurium*, *Shigella flexneri*, *Proteus vulgaris*, *Enterococcus faecalis*, *Pseudomonas aeruginosa*, and *Salmonella typhi-B* [53]. It was reported that phthalic acid increased the superoxide production and ROS generated oxidative stress in bacterial cytoplasm, which eventually led to the death of cell. In addition, it has the ability to interfere with quorum sensing mediated virulence factors [52, 54].

Myristic acid also showed potent antibacterial potential against *Listeria monocytogenes* and it acts on the bacterial cell wall, membrane permeability, and also causes changes in genomic DNA, which might result the cell death [55]. The cytoplasmic membrane is certainly the target point, attacked by long-chain fatty acids (myristic acid) for killing the pathogens [56]. Moreover, the antibacterial mechanism of apigenin are: damage the cytoplasmic membrane and inhibition of nucleic acids synthesis [57] and it was previously reported that mangiferin also showed the inhibitory potential against *B*. *pumilus*, *B*. *cereus* and *Salmonella virchow* [58].

Oleandomycin, is a macrolide antibiotic and found in F23 fraction, its biosynthesis was also previously reported from *Streptomyces antibioticus* [59] but this is first claim for biosynthesis of oleandomycin in *Loig*. *coryniformis*. This antibiotic binds to ribosomal nascent peptide exit tunnel, adjacent to the peptidyl transferase centre, and prevents protein biosynthesis [60]. The F23 fraction was compared with commercially available oleandomycin antibiotic (Fig 5), and the comparison of 23 fraction with commercial oleandomycin depicted the presence of other bioactive metabolites *i*.*e*., phthalic acid, myristic acid, Mangiferin, 16-hydroxylpalmatic acid and apigenin in addition to oleandomycin. The activities of identified metabolites in bioactive fractions (F23) of *Loig*. *coryniformis* BCH-4 reported in this research had already been reported as antibacterial metabolites from other microbial sources. However, there has been no previous data available about these metabolites present in *Loig*. *coryniformis*.

Metabolites with antibacterial potential follow many mechanisms for disabling bacteria. Many of these metabolites target the key components of bacterial metabolism including inhibition of cytoplasmic membrane, inhibition of nucleic acids synthesis and DNA damage [61]. Consequently, in current study, identified metabolites were analysed through in silico, molecular docking to investigate their binding pattern with dihydrofolate reductase of *S*. *aureus*, while DNA polymerase III alpha subunit of *E*. *coli* and putative deacetylase BC1534 of *B*. *cereus* (Figs 6 and 7, and S5-S8 Figs in S1 File).

Primarily, the enzyme dihydrofolate reductase (DHFR) is involved in the pathway of folic acid. This enzyme reduces dihydrofolate to tetrahydrofolate, thus promoting biosynthesis of thymidylate. Moreover, it also improving the DNA translation, RNA transcription, protein replication, and controlling cell proliferation [62]. Secondly, a multi-subunits enzyme, DNA polymerase III (Pol III) responsible for the replication of bacterial genome, with actual DNA synthesis, carried out by Pol III α subunit [63]. While putative deacetylase BC1534 protein of *B*. *cereus* is an enzyme which exhibits deacetylase activity with the *N*-acetyl moiety of the *N*-acetylglucosamine, diacetylchitobiose, and triacetylchitotriose [64]. Since these selected enzymes are pivotal for bacterial survival, these are as the key targets of antibacterial agents [62, 63]. Oleandomycin being macrolide antibiotic inhibits protein synthesis by binding to the 50s subunit of ribosome by interfering with translocation of amino acids to protein synthesis machinery during translation. But in the current study oleandomycin showed substantial binding interactions with selected receptor proteins with good docking score and RMSD value. Besides, another macrolide, erythromycin which also inhibits protein synthesis by binding with 50s subunit of ribosome but when docked to spike protein of SARS-CoV-2 revealed good binding potential with binding energy of -5.8 kcal/mol [65]. Similarly, two macrolides (i.e., oleandomycin and erythromycin) were docked to efflux pumps AcrB and MexB to predict their binding interactions and potential role as efflux inhibitors in Gram-negative bacteria [66]. Hence oleandomysin could form good binding interactions with selected proteins (i.e., DNA polymerase III α-subunit, putative deacetylase BC1534, and dihydrofolate reductase) to inhibit bacterial growth. All the identified metabolites of this study exhibited well established bonds with one or more amino acids in the active pocket of the enzymes. Overall, in silico docking analysis of the identified metabolites matches with in vitro analysis and it was revealed that all the identified metabolites have participated in in vitro antibacterial potential.

## Conclusions

In the current study, the column-fraction (F23) of *Loigolactobacillus coryniformis* BCH-4 (KX388387), demonstrated potent antibacterial and antibiofilm activity against pathogenic bacteria (*E*. *coli*, *B*. *cereus*, and *S*. *aureus*). The bioactive fraction was employed to tandem mass spectrometry and leading to the identification of phthalic acid, myristic acid, mangiferin, 16-hydroxylpalmatic acid, apigenin and a macrolide antibiotic, oleandomycin. Moreover, Ligand- protein interaction via docking also validated the potential inhibitory activity of these 6 metabolites. Thus, the identified metabolites from *Loigolactobacillus coryniformis* BCH-4 with antibacterial activity are potential candidates for drug development.

## Supporting information

S1 File (DOCX)
