# Supplementary material for: Agonistic antibacterial potential of Loigolactobacillus coryniformis BCH-4 metabolites against selected human pathogenic bacteria: An in vitro and in silico approach
Source: PLoS One. 2023 Aug 10;18(8):e0289723. doi: 10.1371/journal.pone.0289723 (PMC10414564; doi:10.1371/journal.pone.0289723)
Supplement: S1 File — (DOCX) [file pone.0289723.s001.docx]

**Agonistic Antibacterial Potential of *Loigolactobacillus coryniformis* BCH-4 Metabolites Against Selected Human Pathogenic Bacteria: An In Vitro and In Silico Approaches**

**Supplementary material**

Fig S 1. ESI-MS^2^ of Phthalic acid (*m/z* 165.1) @CID 2.6, in negative ion mode

Fig S 2. ESI-MS^2^ of Myristic acid (*m/*z 227.4) @CID 3.7, in negative ion mode

Fig S 3. ESI-MS^2^ of 16-hydroxylpalmatic acid (*m/z* 271.2) @CID 3.9, in negative ion mode

Fig S 4. ESI-MS^2^ of Apigenin (*m/z* 269.1) @CID 3.8, in negative ion mode

**(a)**

| 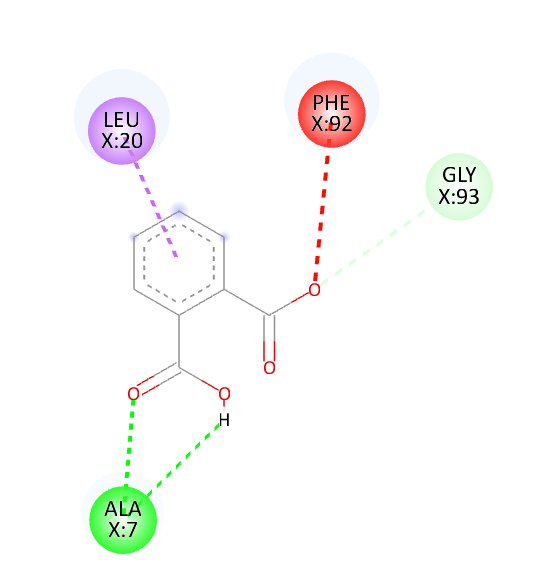  **(aꞌ)** | 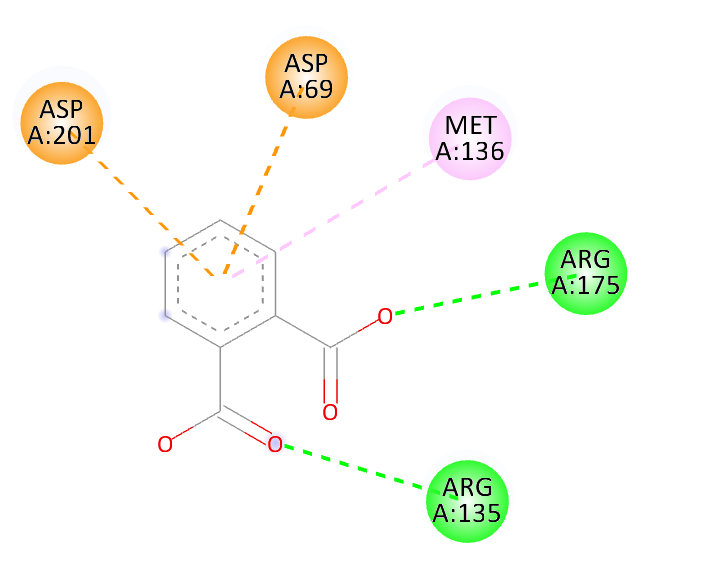  **(bꞌ)**  **(b)** | 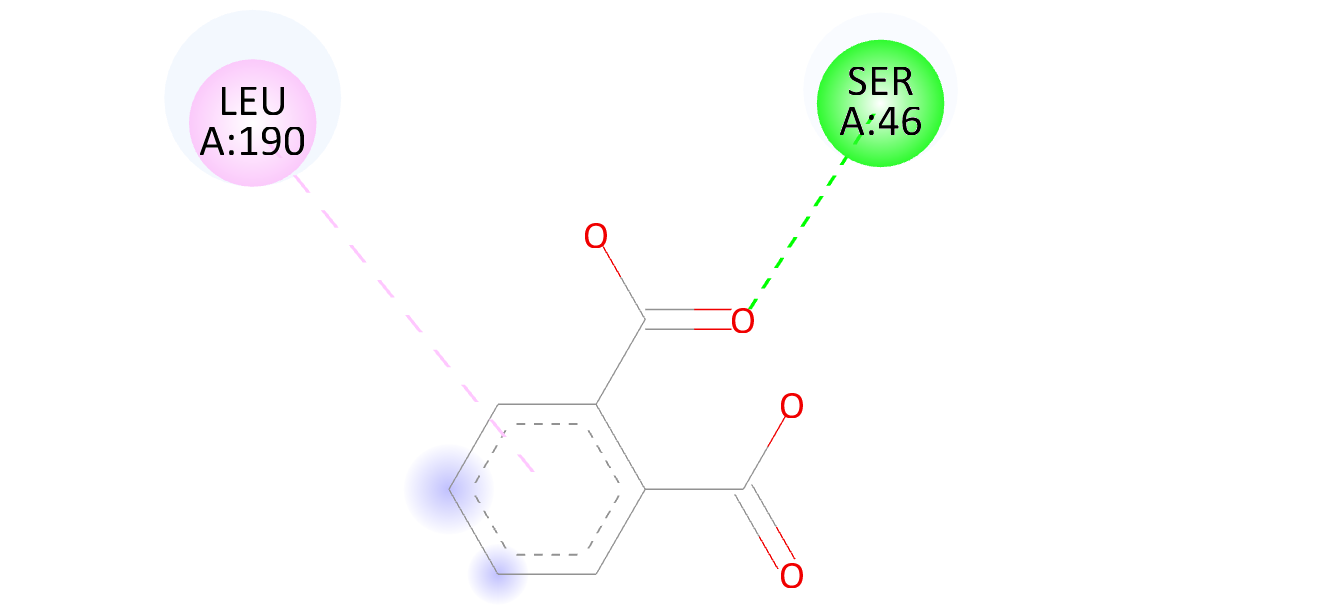  **(cꞌ)**  **(c)** |
| --- | --- | --- |
| 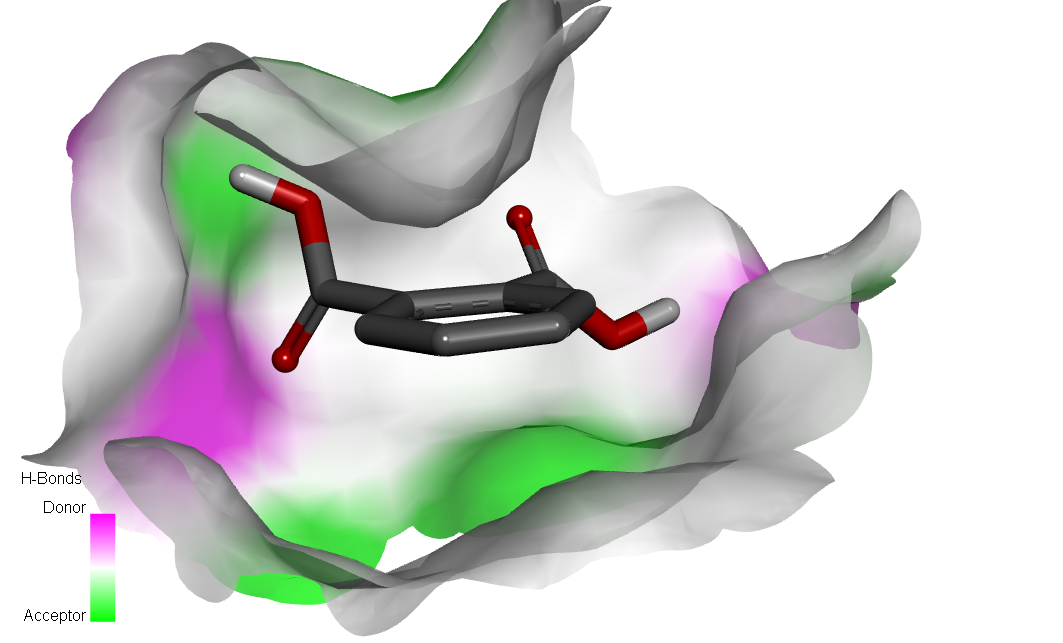 | 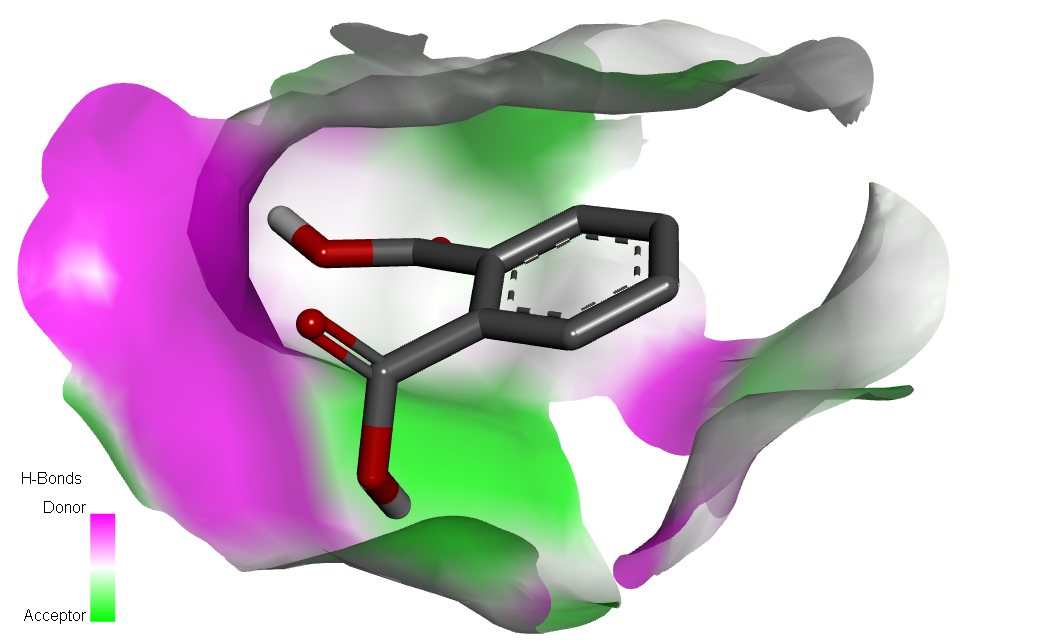 | 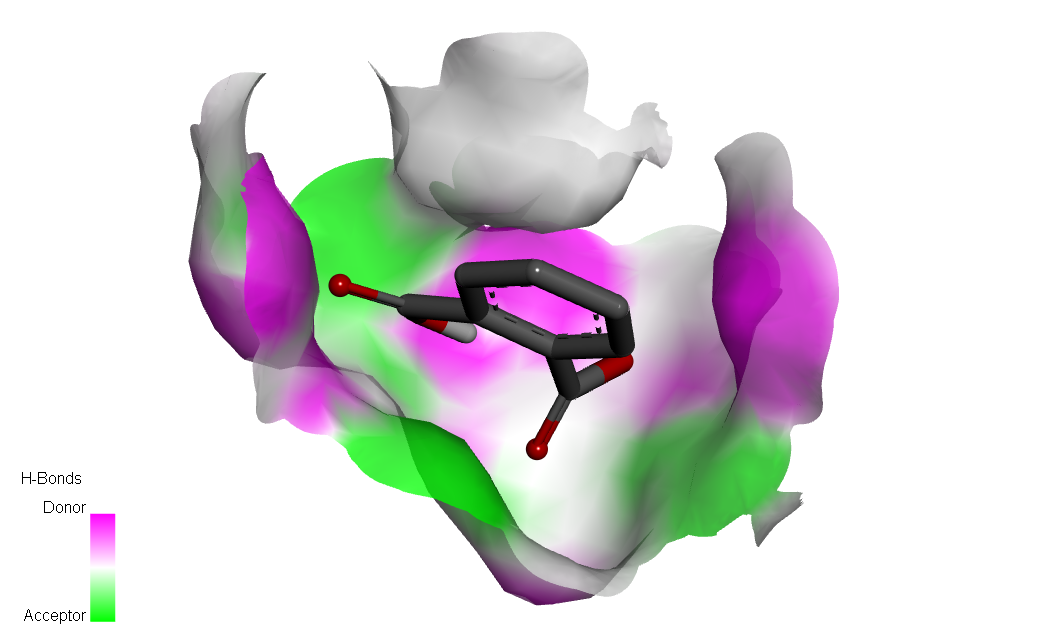 |

Fig S 5. Interactions (a-c) and binding patterns (a'-c') of Phthalic acid with different receptor proteins. (a, a') dihydrofolate reductase from *S. aureus* (b, b') DNA polymerase III α-subunit from *E. coli* (c, c') putative deacetylase BC1534 from *B. cereus.*

| 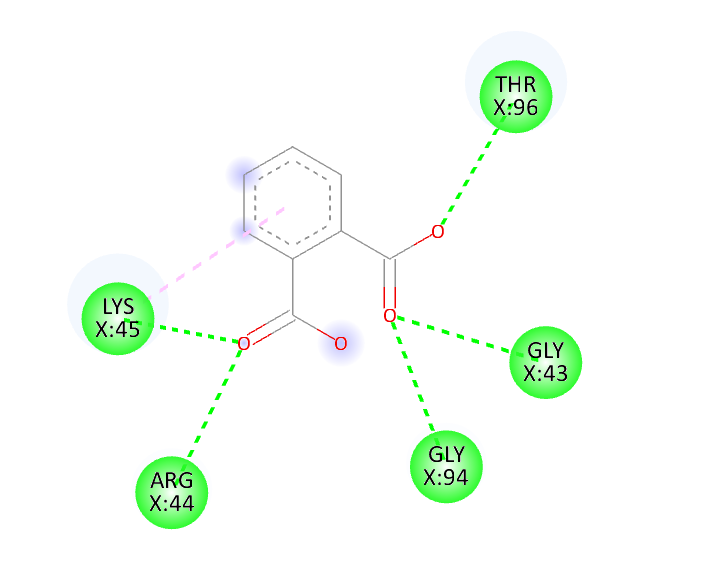  **(aꞌ)**  **(a)**  **(bꞌ)** | 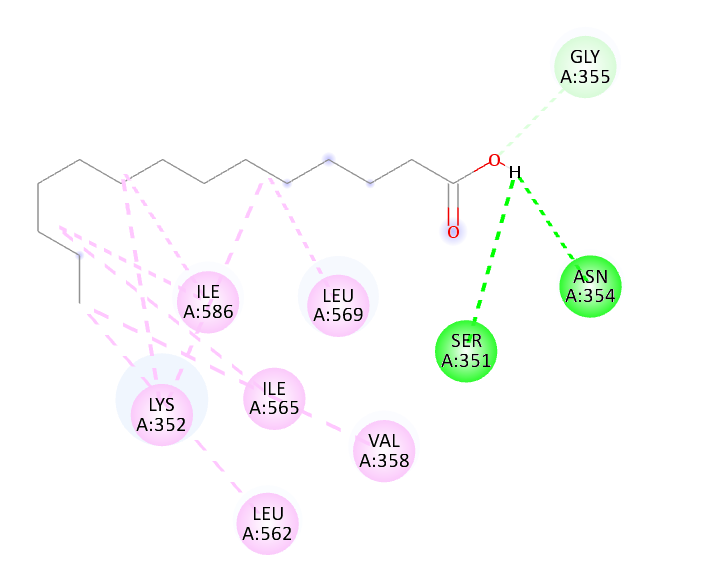  **(cꞌ)**  **(b)** | 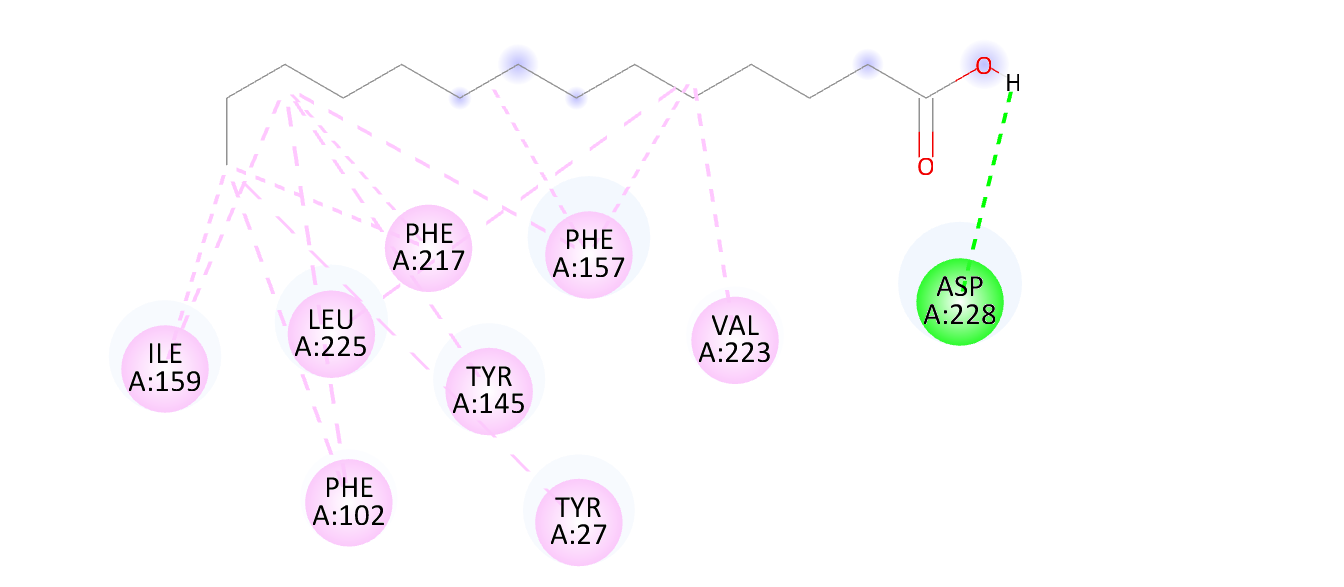  **(c)** |
| --- | --- | --- |
| 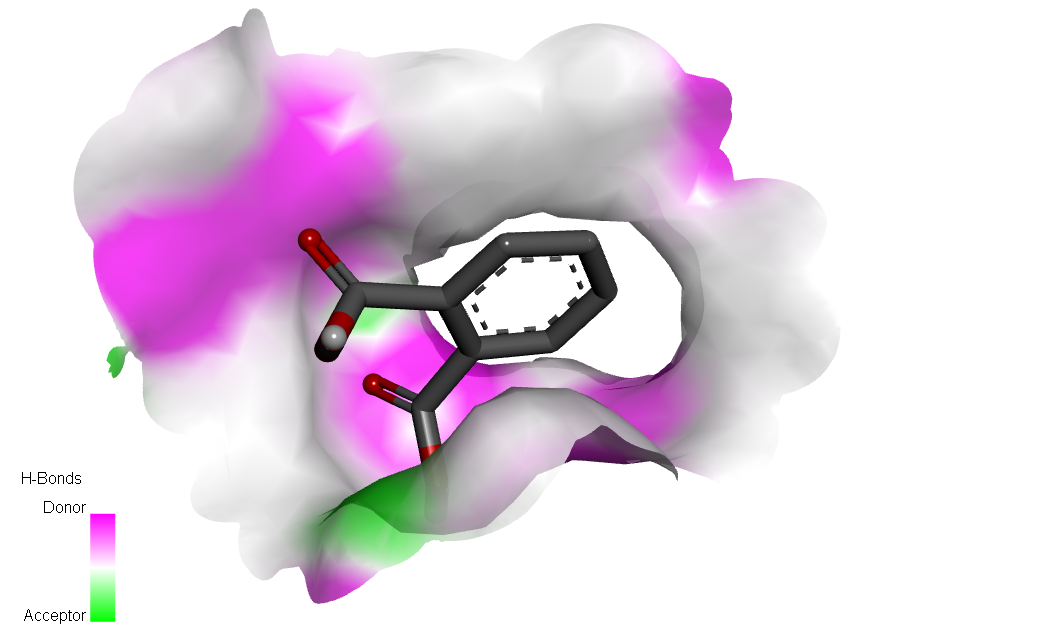 | 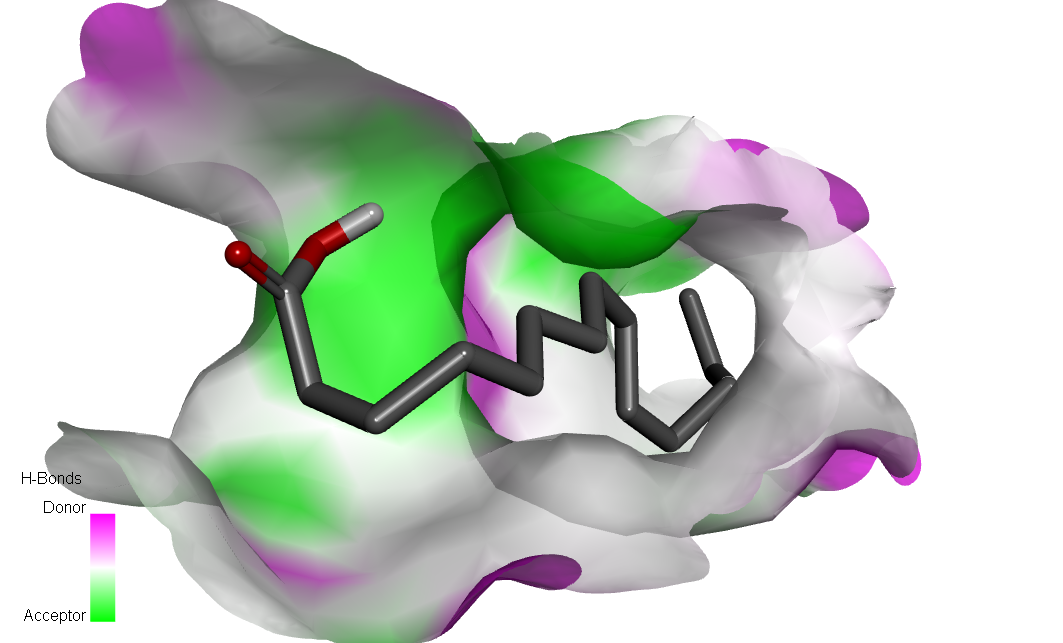 | 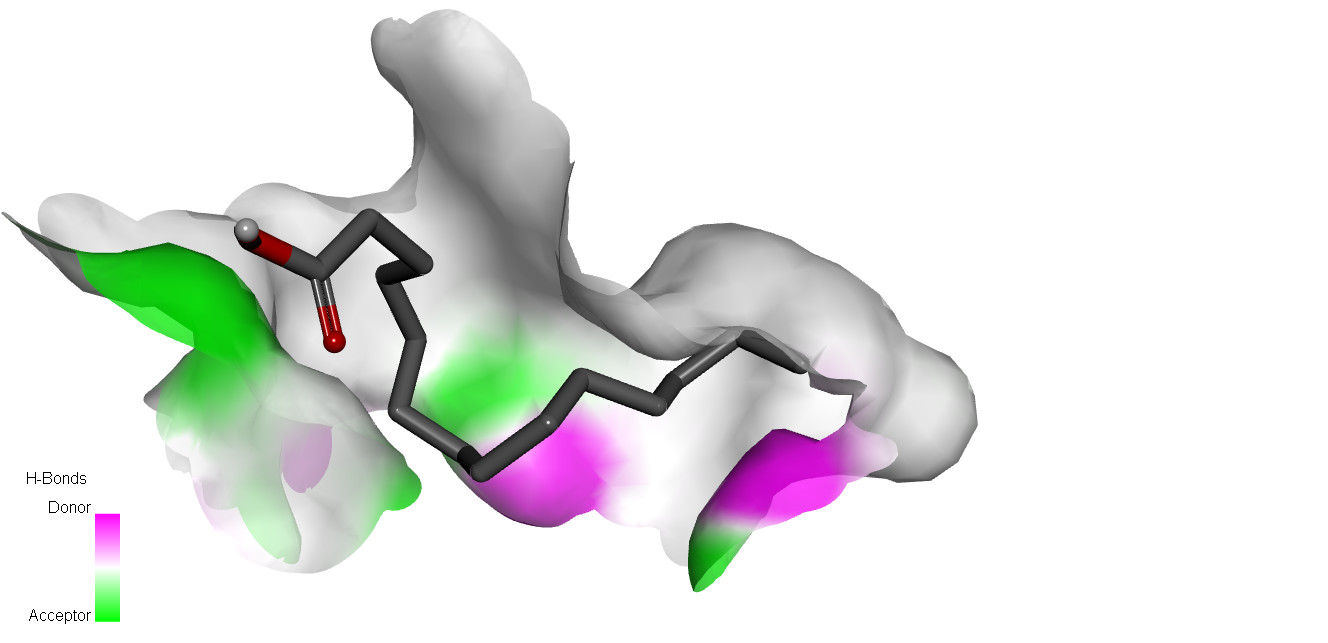 |

Fig S 6. Interactions (a-c) and binding patterns (a'-c') of Myristic acid with different receptor proteins. (a, a') dihydrofolate reductase from *S. aureus* (b, b') DNA polymerase III α-subunit from *E. coli* (c, c') putative deacetylase BC1534 from *B. cereus.*

| 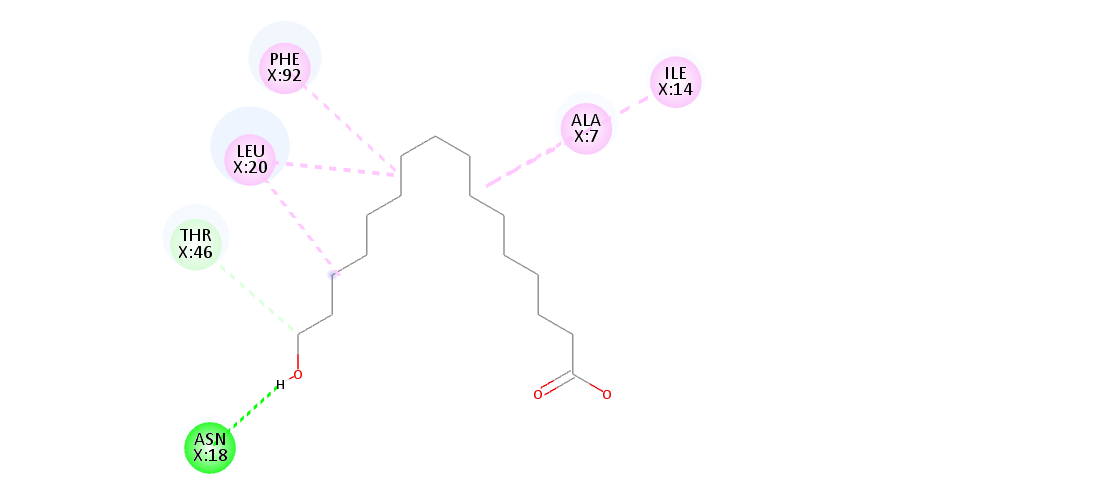  **(aꞌ)**  **(a)**  **(bꞌ)** | 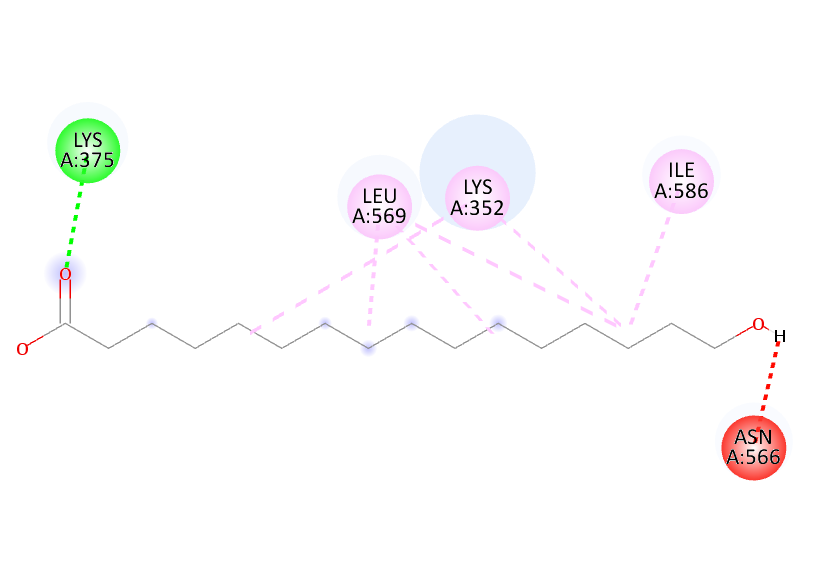  **(c)**  **(b)** | 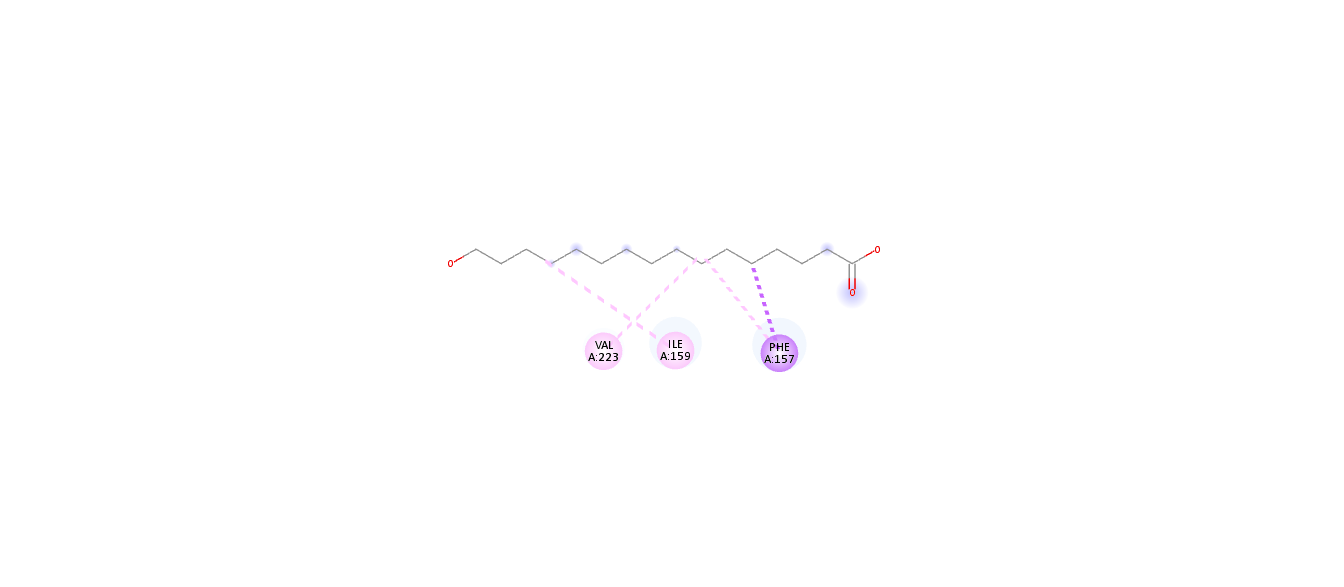  **(cꞌ)** |
| --- | --- | --- |
| 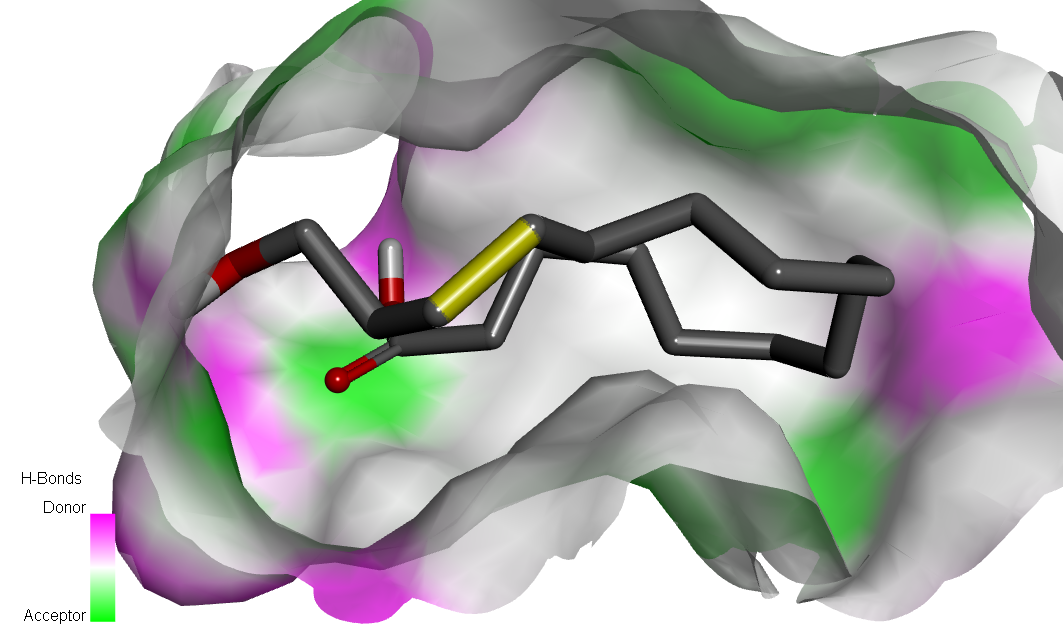 | 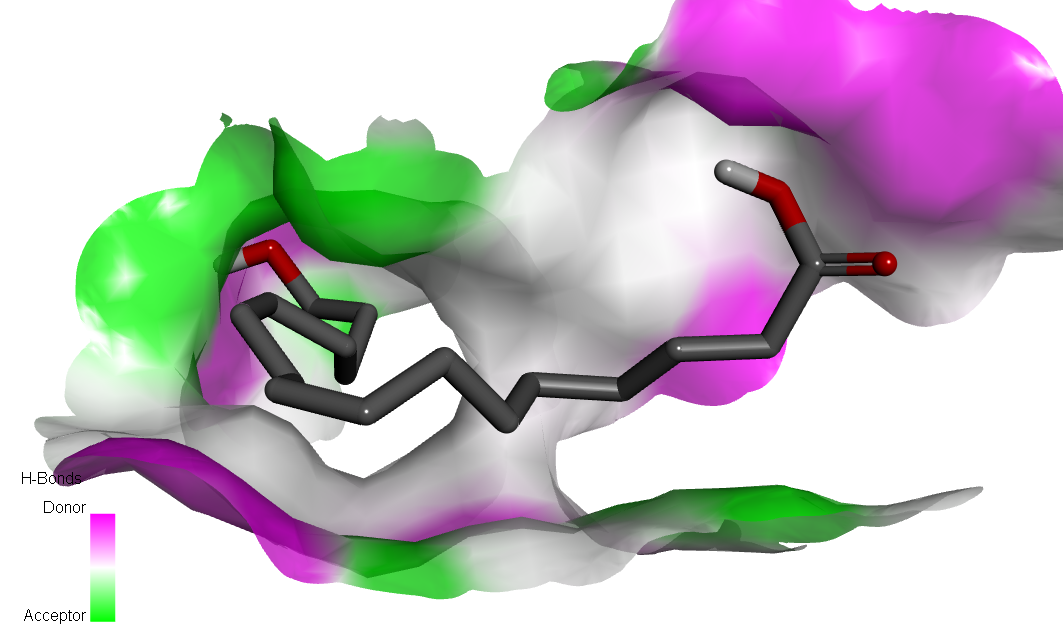 | 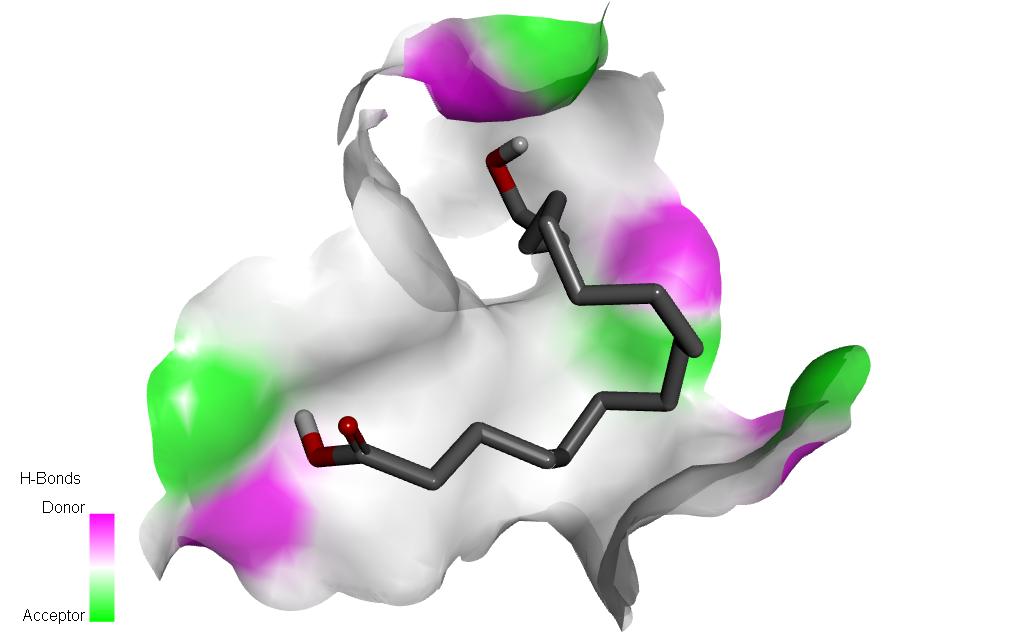 |

Fig S 7. Interactions (a-c) and binding patterns (a'-c') of 16-hydroxypalmitic acid with different receptor proteins. (a, a') dihydrofolate reductase from *S. aureus* (b, b') DNA polymerase III α-subunit from *E. coli* (c, c') putative deacetylase BC1534 from *B. cereus*

| 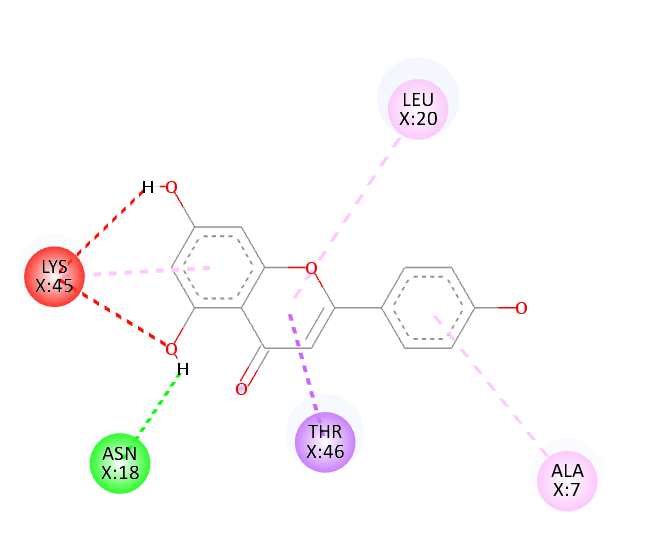  **(a)** | 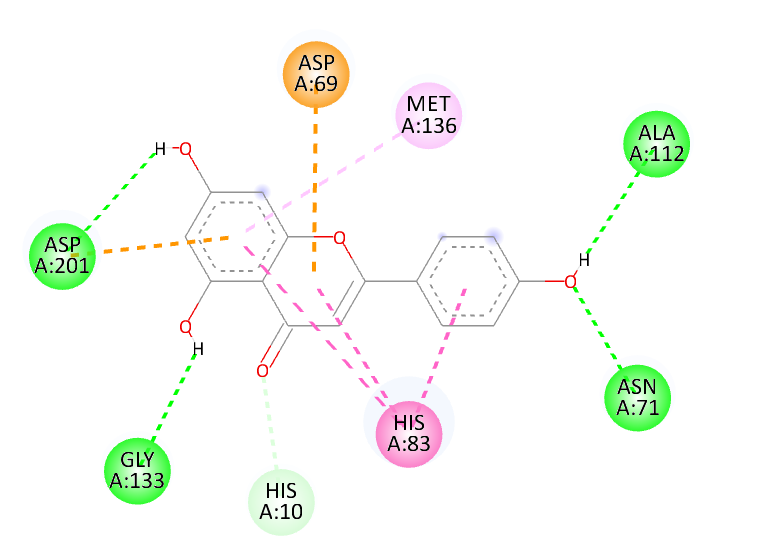  **(b)** | 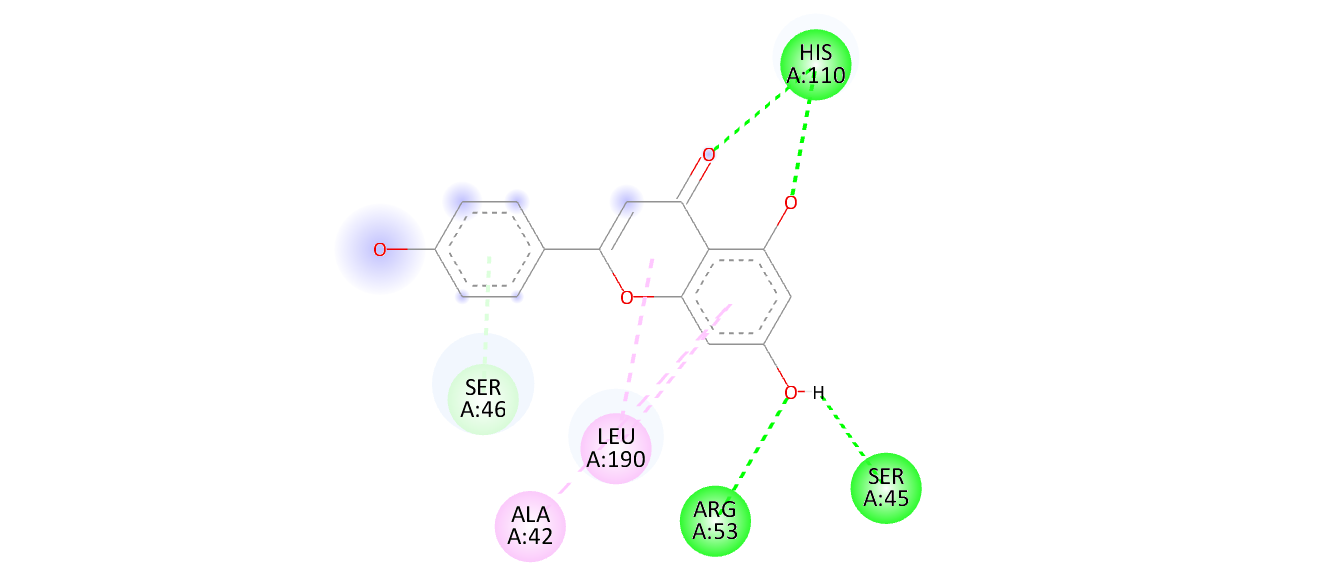  **(c)** |
| --- | --- | --- |
| 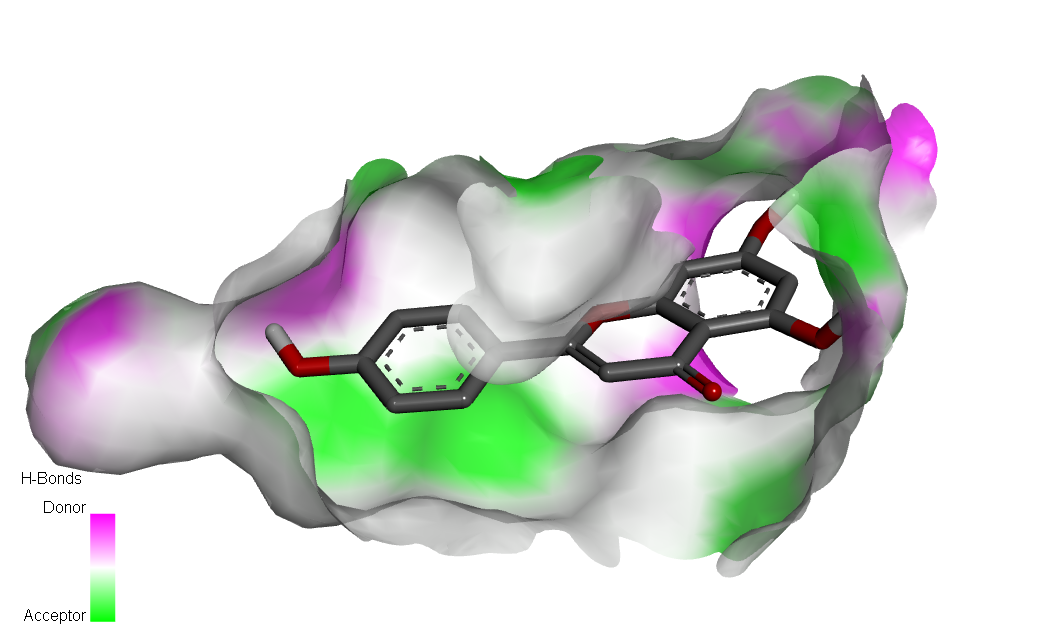  **(bꞌ)**  **(aꞌ)** | 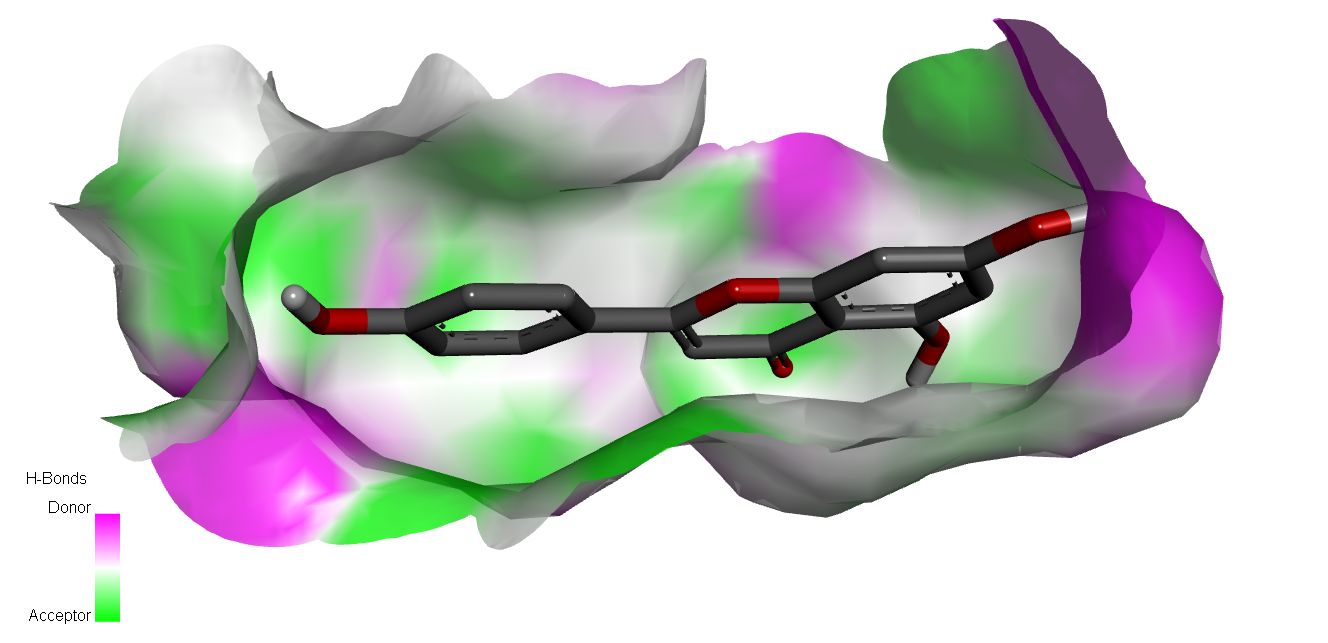  **(cꞌ)** | 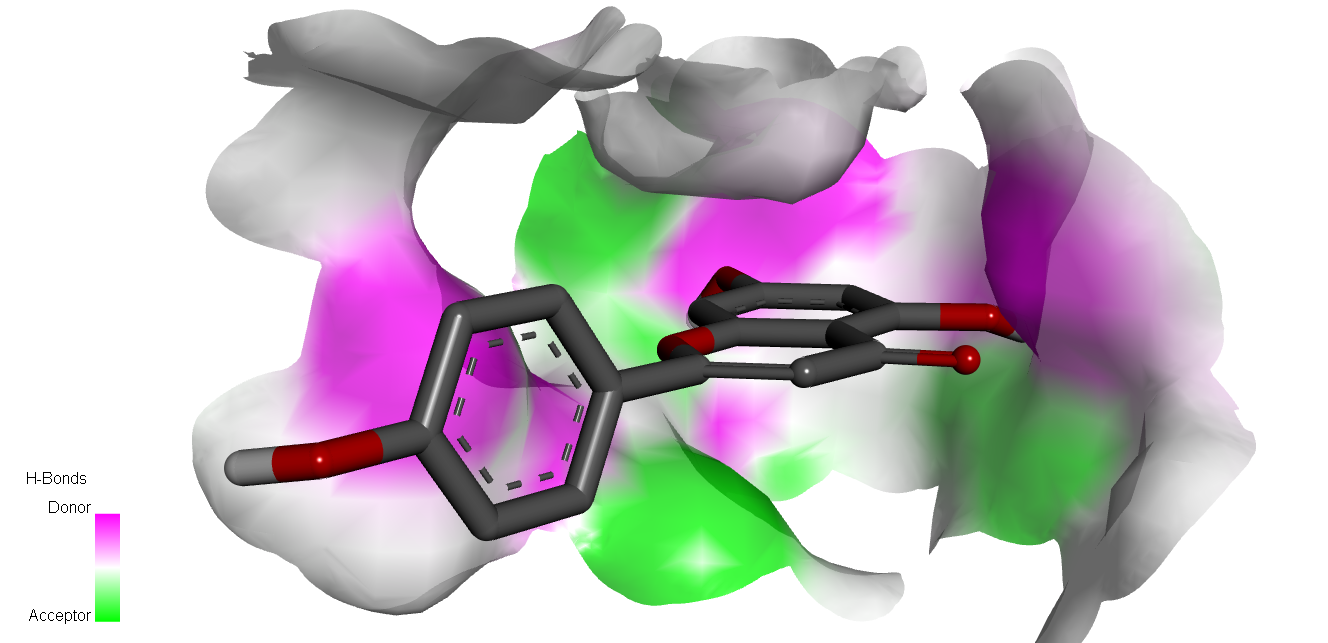 |

Fig S 8. Interactions (a-c) and binding patterns (a'-c') of Apigenin with different receptor proteins. (a, a') dihydrofolate reductase from *S. aureus* (b, b') DNA polymerase III α-subunit from *E. coli* (c, c') putative deacetylase BC1534 from *B. cereus.*

*.*
